# Comparative Gene Expression Profiling of Tobacco-Associated HPV-Positive versus Negative Oral Squamous Carcinoma Cell Lines

**DOI:** 10.7150/ijms.35133

**Published:** 2020-01-01

**Authors:** Silvia Lepore, Giacomo Lettini, Valentina Condelli, Lorenza Sisinni, Annamaria Piscazzi, Vittorio Simeon, Pietro Zoppoli, Maria Carmela Pedicillo, Maria Iole Natalicchio, Michele Pietrafesa, Matteo Landriscina

**Affiliations:** 1 Laboratory of Pre-Clinical and Translational Research, IRCCS, Referral Cancer Center of Basilicata, Rionero in Vulture, Italy;; 2 Medical Oncology Unit, Department of Medical and Surgical Sciences, University of Foggia, Italy;; 3 Medical Statistics Unit, University of Campania Luigi Vanvitelli, Naples, Italy;; 4 Anatomic Pathology Unit, Department of Clinic and Experimental Medicine; University of Foggia, Italy;; 5 Section of Clinic Pathology, Riuniti Hospital, Foggia, Italy

**Keywords:** oral squamous carcinoma, Wnt/βCatenin pathway, stemness, tobacco

## Abstract

**Background:** HPV-positive oral squamous cell carcinomas (OSCCs) are specific biological and clinical entities, characterized by a more favorable prognosis compared to HPV-negative OSCCs and occurring generally in non-smoking and non-drinking younger individuals. However, poor information is available on the molecular and the clinical behavior of HPV-positive oral cancers occurring in smoking/drinking subjects. Thus, this study was designed to compare, at molecular level, two OSCC cell lines, both derived from drinking and smoking individuals and differing for presence/absence of HPV infection.

**Methods:** HPV-negative UPCI-SCC-131 and HPV16-positive UPCI-SCC-154 cell lines were compared by whole genome gene expression profiling and subsequently studied for activation of Wnt/βCatenin signaling pathway by the expression of several Wnt-target genes, βCatenin intracellular localization, stem cell features and miRNA let-7e. Gene expression data were validated in head and neck squamous cell carcinoma (HNSCC) public datasets.

**Results:** Gene expression analysis identified Wnt/βCatenin pathway as the unique signaling pathway more active in HPV-negative compared to HPV-positive OSCC cells and this observation was confirmed upon evaluation of several Wnt-target genes (i.e., *Cyclin D1, Cdh1, Cdkn2a, Cd44, Axin2, c-Myc* and* Tcf1*). Interestingly, HPV-negative OSCC cells showed higher levels of total βCatenin and its active form, increase of its nuclear accumulation and more prominent stem cell traits. Furthermore, miRNA let-7e was identified as potential upstream regulator responsible for the downregulation of Wnt/βCatenin signaling cascade since its silencing in UPCI-SCC-154 cell resulted in upregulation of Wnt-target genes. Finally, the analysis of two independent gene expression public datasets of human HNSCC cell lines and tumors confirmed that Wnt/βCatenin pathway is more active in HPV-negative compared to HPV-positive tumors derived from individuals with smoking habit.

**Conclusions:** These data suggest that lack of HPV infection is associated with more prominent activation of Wnt/βCatenin signaling pathway and gain of stem-like traits in tobacco-related OSCCs.

## Background

Oral squamous cell carcinoma (OSCC) is responsible for 1.8% of cancer mortality worldwide with 300.000 new cases every years and is associated to tobacco use, unhealthy diet, excessive alcohol consumption and HPV infection 1*.* Recent evidences suggest that HPV-positive OSCCs are characterized by a different clinical, molecular and biological behavior compared to HPV-negative cancers. Indeed, the majority of HPV-positive OSCCs have better prognosis and occur in non-smoking, non-drinking and younger individuals than HPV-negative OSCCs. The reason of this better survival is likely related to the different population-affected profile and lack of field cancerization 2. However, a subset of HPV-related OSCCs, up to one-third of cases, occurs in smoking and drinking subjects, this representing a subgroup of tumors with unclear clinical and biological characters 3. Indeed, recent evidence suggests that cigarette smoking changes the clinical behavior of HPV-positive OSCCs, being responsible for reduced responsiveness to therapies and worsening of their prognosis 4.

Several molecular and epidemiological studies showed relevant differences in terms of genome-wide gene expression profiles between HPV-positive and HPV-negative OSCCs, these likely influencing clinical outcomes 5-6. Indeed, Holzinger et al. identified a different protein expression pattern between HPV-positive and HPV-negative OSCCs, with HPV-positive tumors characterized by high p16 expression, lower levels of pRb and Cyclin D1 and normal p53 level 7. In addition, a number of studies identified DNA replication, cell cycle regulation and DNA repair as prominent cell functions associated to genes differentially expressed in HPV-positive versus HPV-negative cancers 8-9. Since i) a broad understanding of the molecular differences between different subtypes of OSCCs represents an important step in the development of personalized treatments and ii) poor information is available on the role of HPV infection in tobacco-related oral squamous carcinomas, this study was designed to evaluate the gene expression profile of two OSCCs cell lines derived from smoking and drinking patients and differing for presence/absence of HPV infection and to validate these profiles in public datasets. Our data suggest that lack of HPV infection is associated with a more prominent activation of Wnt/βCatenin pathway and gain of stem-like traits.

## Materials and Methods

### Cell lines and siRNA transfection

OSCC UPCI-SCC-131 and UPCI-SCC-154 cell lines (DSMZ**,** Braunschweig Germany) were cultured at 37 °C in a humidified atmosphere of 5% CO_2_ in DMEM (Gibco, Life Technologies, Carlsbad, CA, USA) supplemented with 10% (v/v) fetal bovine serum (FBS, Gibco, Life Technologies, Carlsbad, CA, USA) and 1% (v/v) penicillin/streptomycin (Gibco, Life Technologies, Carlsbad, CA, USA).

miRNA let-7e was silenced using 60 nM mirVana miRNA Let-7e-5p inhibitor (Ambion, Thermo Fisher Scientific, Waltham, Massachusetts, USA, Catalog. 4464084, ID: MH12304). Negative mirVana inhibitor (Ambion, Thermo Fisher Scientific, Waltham, Massachusetts, USA, Catalog. 464076) was used as negative control. Transfection was performed with Lipofectamine RNAiMAX (Invitrogen, Thermo Fisher Scientific, Waltham, Massachusetts, USA,) according to the manufacturer's instructions.

### Immunoblot analysis

Cells were lysed with RIPA buffer containing 25mM Tris HCl pH 7.4, 150mM NaCl, 1% (v/v) NP-40, 1% (w/v) sodium deoxycholate, 0.1% (w/v) SDS for 30 min on ice. Cell lysates were centrifuged at 1200 rpm for 10 minute at 4°C and supernatants assayed for protein concentration by Bradford method. Thirty micrograms of total proteins were loaded onto ready-to-use 4-20% polyacrylamide gels (Bio-Rad, Hercules, CA, USA), separated by electrophoresis and transferred onto nitrocellulose membranes (Trans-Blot, Bio-Rad, Hercules, CA, USA). The following primary antibodies were used: mouse monoclonal anti-active-βCatenin (cod.8814), rabbit polyclonal anti-βCatenin (cod.9562), rabbit monoclonal anti-Cyclin D1 (cod.92G2), rabbit monoclonal anti-cMyc (D84C12)XP (cod. 5605), rabbit monoclonal anti-Tcf-1 (C63D9) (cod.2203) from Cell Signaling Technology (Cell Signaling, Boston, MA, USA); mouse monoclonal anti-E-Cadherin (cod.610181) from BD (Becton Dickinson, BD, Franklin, NJ, USA); rabbit monoclonal anti-Axin-2 (cod. ab109307) from Abcam (Abcam, Cambridge, UK); rabbit polyclonal anti-Lgr5 (H-76) (cod. sc-135238), rabbit polyclonal anti-GAPDH (cod. sc-47724) and rabbit polyclonal anti-βActin (cod. sc-47778) from Santa Cruz Biotechnology. Specific proteins were reveled using the enhanced chemiluminescence (ECL) reagent (Bio-Rad, Hercules, CA, USA). GAPDH or β-Actin were detected as loading controls.

### Clonogenic assay

Single cell suspension of 1000 cells were seeded on 60 mm tissue culture dishes and cultured for 15 days with medium changes every 3 days. Colonies were fixed with 4% paraformaldehyde at room temperature for 10 minutes and, after removing the fixation solution, stained with crystal violet solution for 20 minutes, rinsed with H_2_O and air-dried at room temperature. Colonies were counted under a light microscope and numbers used as index for clonogenicity.

### Flow-cytometry

Non-confluent cell cultures were trypsinized into single cell suspension, washed with phosphate buffered saline (PBS) and counted. 1.0x10^6^ cells were incubated with APC-conjugated mouse monoclonal Anti-Human CD44 (Becton Dickinson, BD, Franklin, NJ, USA) or mouse IgG2b κ Isotype Control (Becton Dickinson, BD, Franklin, NJ, USA), used as negative control. After washing twice with PBS, samples were analyzed by FACSb Calibur (Becton Dickinson, San Jose, CA, USA).

### Immunocytochemistry

Cells were grown on glass slides at a density of 5x10^3^ cells, rinsed with PBS, fixed in alcohol, rehydrated in graded ethanol solution and finally washed for 5 minutes with distilled water. p16 expression was assessed by standard linked streptavidin-biotin horseradish peroxidase technique (LSAB-HRP) using a specific mouse monoclonal primary antibody produced against the p16 INK4a protein (CINtec p16 Histology, clone E6H4, Ventana) delivered by the Benchmark XT autostainer (Ventana Medical Systems Inc, Tucson, AZ) in combination with Ventana detection kits and accessories. Immunocytochemical staining was evaluated using the Olympus BX41 microscope.

### Immunofluorescence and confocal microscopy analysis

15x10^3^ cells were seeded on chamber slides and cultured for 48 hours. Cells were fixed with 4% (w/v) paraformaldehyde in PBS at room temperature for 15 minutes and permeabilized with 0.1% (v/v) Triton-X100 (Sigma-Aldrich, Milan, Italy) for 10 minutes at room temperature. Non-specific binding sites were blocked by incubation in 1% (w/v) bovine serum albumin (BSA) in PBS for 30 minutes at room temperature. Cells were incubated with mouse monoclonal anti-active-βCatenin (1:400, Cell Signaling Technology, Boston, MA, USA) overnight at 4°C and subsequently with FITC-anti-mouse IgG antibody (1:1000, Sigma-Aldrich, Milan, Italy) for 1 hour at room temperature. Nuclei were counterstained with 4',6-diamidino-2-phenylindole (DAPI) using VECTASHIELD® Mounting Medium (Biorad, Hercules, CA, USA) and viewed under a confocal microscope Nikon Eclipse Ti-E microscope equipped with a unique Perfect Focus System (PFS) and coupled to Laser scanning confocal microscope C2 (Nikon Instruments S.p.A, Florence, Italy). Specimens were viewed through a 60X Plan APO oil immersion objective. Digital images were processed using the Nis Elements AR software.

### Multiplex real-time PCR

DNA was extracted by the DNA mini kit (Qiagen, Hilden, Germany) and HPV genotype evaluated by Anyplex II HPV28 assay, according to manufacturer's recommendations (Seegene, Seoul, South Korea). Data recording and interpretation were automated with the Seegene viewer software.

### RNA Extraction and Reverse Transcriptase PCR analysis

Total RNA was extracted using the TRIzol Reagent (Life Technologies, Carlsbad, CA, USA). For first strand synthesis of cDNA, 1 µg of RNA was used in a 20 µl reaction mixture utilizing a Transcriptor First Strand cDNA Synthesis Kit (Roche, Mannheim, Germany). For Real-time PCR analysis, 0.5 ng of cDNA samples were amplified using the LightCycler 480 SYBR Green I Master in a LightCycler 480 (Roche, Basel, Switzerland). The following primers were used: *c-Myc* forward 5'-TTCGGGTAGTGGAAAACCAG-3', reverse 5'-CAGCAGCTCGAATTTCTTCC-3' (PCR product 203 bp); *Cyclin D1* forward 5'-CTACTACCGCCTCACACGCTT-3', reverse 5'-AGCCCTGGAGTCAAGCC-3' (PCR product 198 bp); *Axin2* forward 5'-AGGTTCTGGCTATGTCTTTG-3', reverse 5'-AAATGAGGTAGAGACACTTGG-3' (PCR product 201 bp). PCR reaction conditions were as follows: pre-incubation at 95°C for 5 min, followed by 45 cycles of 10 s at 95°C, 10 s at 60°C, 10 s at 72°C. β-Actin was chosen as an internal control.

For miRNA let-7e quantification, RT-PCR was performed in UPCI-SCC-131 and UPCI-SCC-154 cell lines and in UPCI-SCC-154 upon miRNA let-7e silencing. cDNA was synthetized by reverse transcription using the TaqMan™ MicroRNA reverse transcription kit (Applied Biosystems, Monza, Italy), according to the manufacturer's instructions. Real-time PCR analysis was performed using the TaqMan™ MicroRNA assay with specific primer sets (probe has-miR-let-7e-5p, ID 002406, Applied Biosystems, Monza, Italy). The expression of RNU6B was used as internal control (probe RNU6B, ID 001093, Applied Biosystems, Monza, Italy) and the results were calculated using the ∆∆CT (where CT is threshold cycle) method.

### Microarray experiments and data analysis

RNAs were isolated by Trizol method, using three replicates for each cell lines, and evaluated for quality and integrity by the 2100 Bioanalyzer (Agilent Technologies, Waldbronn, Germany). Gene expression analysis was performed using the Illumina TotalPrep RNA (Ambion, Thermo Fisher Scientific, Waltham, Massachusetts, USA) for amplification, labeling and generation of cRNA, while hybridization by Microarray HumanHT-12 v4 Expression BeadChip (Illumina Inc., San Diego, CA, USA) according to the manufacturer's instructions. BeadChip was dried and scanned with an Illumina HiScanSQ system (Illumina Inc., San Diego, CA, USA). Raw data were obtained using Genome Studio Software (Illumina Inc., San Diego, CA, USA). Briefly, quantile normalization algorithm was applied on the data set to correct systematic errors: values below a detection score of 0.05 were filtered out and missing values were imputed. Differential expression analyses were conducted using GenomeStudio Software (Illumina Inc., San Diego, CA, USA) and differently expressed genes (DEGs) were selected with differential score (DiffScore) cutoff set at ±18 (p<0.01). Microarray data were submitted to Array Express under accession number E-MTAB-6152.

Differentially expressed transcripts were displayed using a classic Heatmap, with chromatic variations that reflect the different levels of gene expression between the two cell lines. The heatmap was generated using normalized gene expression values with -1≤log≥1 fold change. The DEGs list was used to evaluate the functional behavior in terms of Biological Processes, performing an enrichment analysis with IPA software (Ingenuity Systems, Redwood City, CA; http://www.ingenuity.com).

### MTT assay

Cell viability was evaluated using the dimethylthiazoldiphenyltetrazoliumbromide (MTT, Sigma-Aldrich, Milan, Italy) dye assay as previously described 10. Briefly, cells were seeded into 24-well plates (1x10^4^ cells/well) and grown up to 144 hours. Cells were then incubated in presence of 500 µM MTT solution for 3 hours at 37°C to allow MTT metabolism into formazan crystals. The formazan crystals were finally solubilized by adding 200 µl of 0.04 N HCl in isopropanol to each microplate well. Adsorbance at 570 nm was measured using a Bio-Tek microplate reader (model EL-340; BioMetallics, Priceston, NJ). Wells containing only DMEM, FBS and MTT were used as controls. In specific experiments, cell viability was evaluated upon exposure to cisplatin (1mg/ml, Accord Healthcare Italia S.r.l.). Cells were treated with cisplatin (1, 10, and 20 μM) for 48 hours and, then, assayed by MTT, as descripted above.

**Public dataset analysis**

The GEO (Gene Expression Omnibus) portal was used to expand gene expression analysis. Public datasets were selected using 'head and neck cancer' as keyword and results were filtered by selecting only those reporting data about HPV status of cell lines and tumors and the smoking habit of patients. Among all, only two datasets with sufficient information were selected (GSE65858 and GSE52088), the first reporting data on human head and neck squamous cell carcinomas (HNSCCs) 11 and the other on HNSCC cell lines. Geo2R application was used to compare groups by calculating the logFC and the p-value. We defined DEGs genes resulting with -0.26 > logFC>0.26 and p-value <0.05. To assess if Wnt/βCatenin pathway is active in HPV-negative versus HPV-positive tumors/cell lines, a Hypergeometric test was performed on all 39 mSigDB collections 12 reporting the word “wnt” into their denomination; the test was performed on previously obtained up-/down-regulated genes of Wnt/βCatenin pathway and was considered significantly enriched if the adjusted p-value was < 0.05 with fdr correction 13. Pathways significantly enriched in tumors or cell lines datasets were further investigated in the respective non-significant dataset with a more relaxed threshold (p-value <0.05, no correction) to assess, at least, a possible common trend. All the analysis were performed in R 14.

### Statistical analysis

Data were analyzed using Prism5 (GraphPad Software Inc., La Jolla CA). Statistical evaluation of data was performed by t-test. Significant differences were assigned to p value <0.05. Data were presented as means ± standard deviation. All experiments were repeated at least three times.

## Results

### Gene expression profiles of OSCC cell lines

In order to address the biological relevance of HPV infection in OSCCs occurring in smoking and drinking individuals, UPCI-SCC-131 and UPCI-SCC-154 cell lines, both derived from Caucasian male patients affected by a squamous cell carcinoma of the oral cavity and with a clinical history of long-time tobacco smoking and excessive alcohol drinking, were selected for this study. Both cell lines are p53 wild type, UPCI-SCC-131 cells positive for the amplification of chromosomal band 11q13, UPCI-SCC-154 cells positive for HPV infection (Table 1).

**Figure 1:**
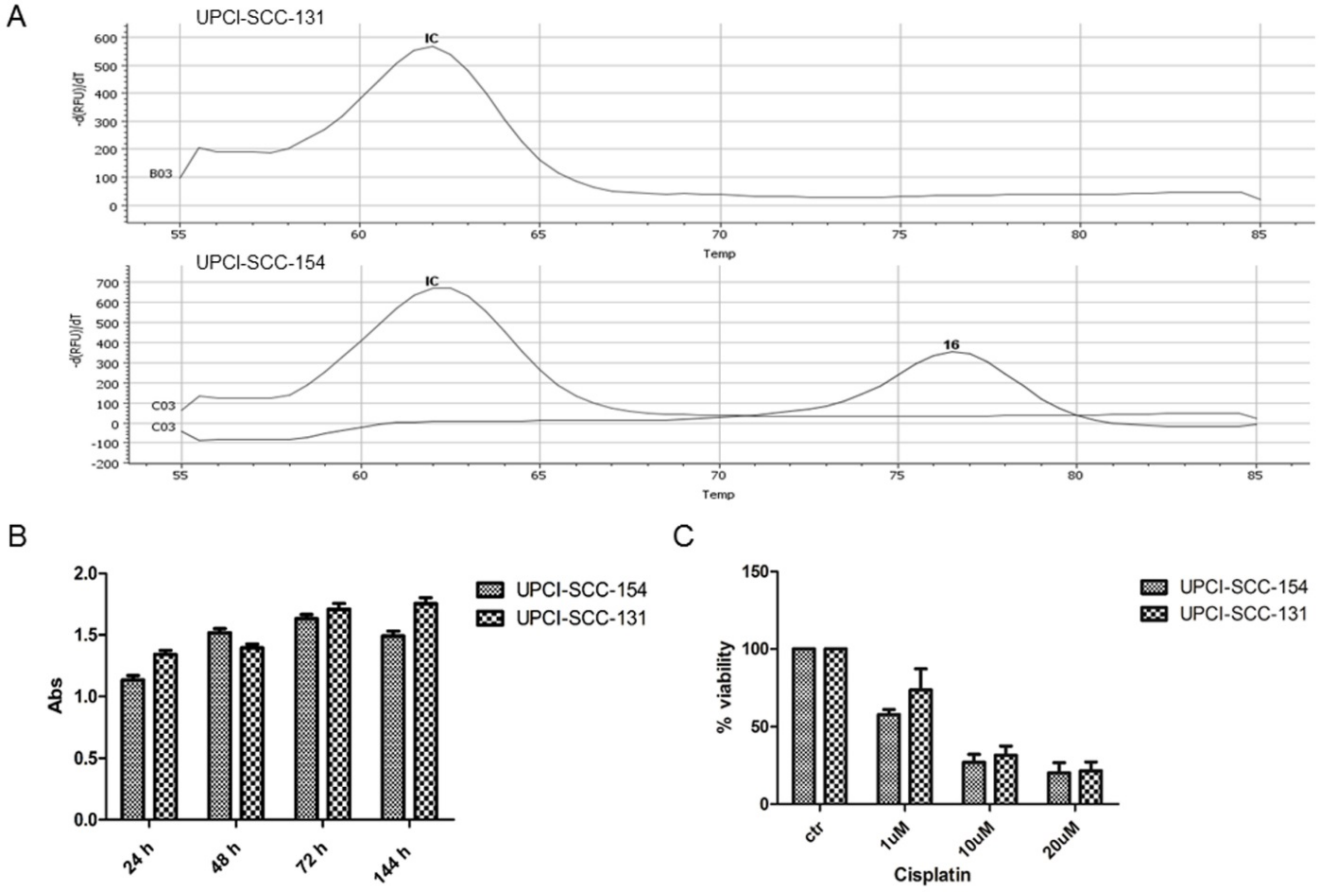
** Characterization of UPCI-SCC-131 and UPCI-SCC-154 cell lines. (A)** HPV-DNA detection in UPCI-SCC-131 and UPCI-SCC-154 cell lines by multiplex RT-PCR. **(B-C)** Comparative analysis of cell proliferation **(B)** and cell viability **(C)** between UPCI-SCC-131 and UPCI-SCC-154 cell lines grown up to 6 days **(B)** or exposed to increasing concentrations of cisplatin for 48 hours **(C)** and evaluated by MTT incorporation.

In preliminary experiments, RT-PCR analysis confirmed the positivity of UPCI-SCC-154 cells for HPV strain 16 infection (Figure 1A). In addition, since HPV-positive OSCC cell lines are generally more resistant to cisplatin cytotoxicity 15, UPCI-SCC-154 and UPCI-SCC-131 cell lines were compared for proliferation rates and response to cisplatin. As reported in Figure 1B-C, no major differences were observed between the two OSCC cell lines in terms of growth rate and sensitivity to cisplatin, this suggesting that UPCI-SCC-154 cells, besides being HPV-positive, behave similarly to HPV-negative UPCI-SCC-131 cells. Thus, in further experiments, UPCI-SCC-154 cells were used as a model of tobacco-related HPV-positive cell line and UPCI-SCC-131 as the HPV-negative counterpart.

**Figure 2:**
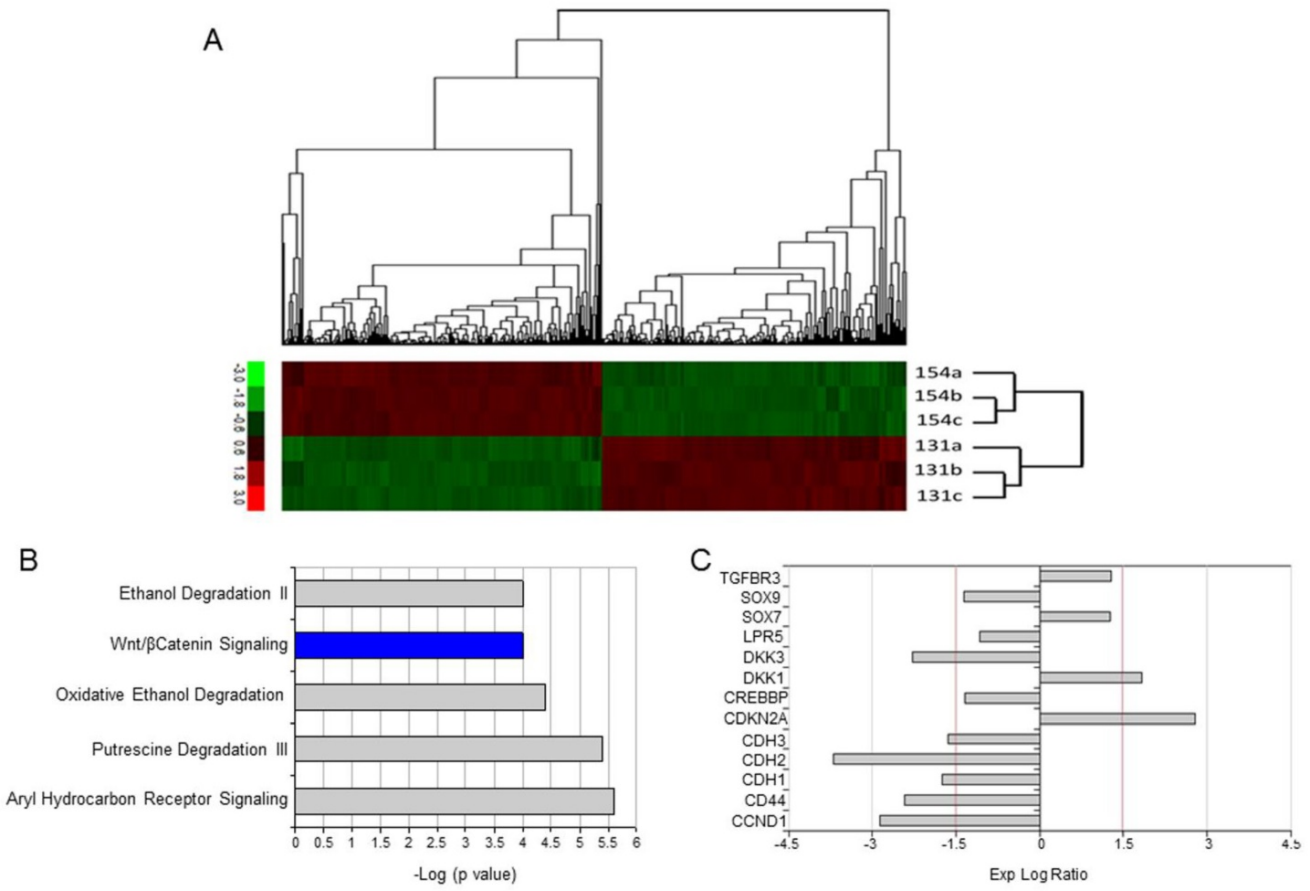
** Differential gene expression profile between UPCI-SCC-131 and UPCI-SCC-154 cell lines. (A)** Heatmap reporting normalized expression values (z score of ΔCT) of significant genes (adjusted p ≤ 0.05, n= 2) differentially expressed between UPCI-SCC-154 and UPCI-SCC-131 cells. The scale ranges from -3 = bright green to +3 = bright red. Each cell line is reported in triplicate. **(B)** List of top canonical pathways according to IPA. Bars represent -log (p-value). Colors represent z-score; in blue are reported pathways with decreased activity while in grey pathways with conflicting or missing data. **(C)** List of 13 Wnt-related/target differentially expressed genes between UPCI-SCC-154 and UPCI-SCC-131 cells. Bar Graphs represent gene expression log ratios.

In order to identify genes differentially expressed between UPCI-SCC-154 and UPCI-SCC-131 cells, we performed a full genome gene expression profiling analysis by Illumina technology. Four hundred seventy-two genes were shown to be differentially expressed (230 up- and 242 downregulated), as reported in Supplementary Table S1 and Figure 2A. IPA was used to predict main signaling pathways differentially modulated between HPV-positive and HPV-negative OSCC cell lines and the top five canonical pathways are reported in Figure 2B. Interestingly, Wnt/βCatenin pathway was identified as the unique signaling pathway unambiguously less active in HPV-positive compared to HPV-negative OSCC cells, with a negative z-score (Figure 2B). Consistently, 13 genes encoding for regulators or target genes of Wnt/βCatenin pathway were differentially expressed between HPV-positive compared to HPV-negative OSCC cells, 8 of them with a log ratio ≥ ±1.5 (Figure 2C). Noteworthy, the expression profile of the vast majority of these genes is consistent with a reduced activity of Wnt/βCatenin signaling pathway in UPCI-SCC-154 compared to UPCI-SCC-131 cells.

**Figure 3:**
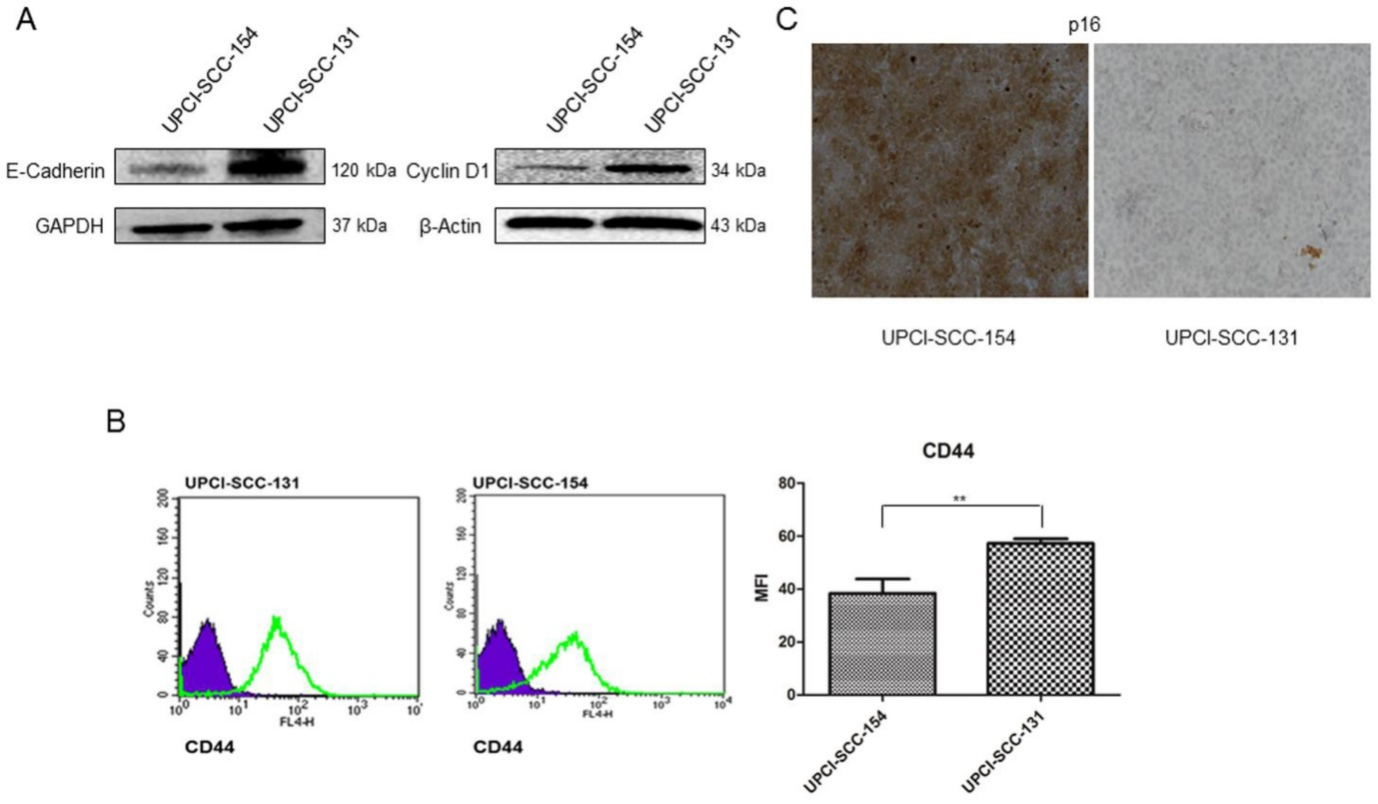
** Validation of Wnt-target genes differentially expressed between UPCI-SCC-131 and UPCI-SCC-154 cells. (A-C)** Analysis of E-Cadherin and Cyclin D1, CD44 and p16 expression in UPCI-SCC-131 and UPCI-SCC-154 cells by, respectively, immunoblot analysis **(A)**, immunocytochemistry **(B)** and flow cytometry **(C)**. **(B)** LSAB-HRP, nuclear counterstaining with haematoxylin; original magnification 10X.** (C)** Histograms represent mean fluorescence intensity (MFI) ±SD. **p value=0.05 respect to UPCI-SCC-131 cells.

To further validate microarray data, the expression of CCND1 (Cyclin D1) and CDH1 (E-Cadherin) was analyzed by immunoblot, CDKN2A (p16) by immunocytochemistry and CD44 by flow-cytometry in both cell lines. As shown in Figure 3, UPCI-SCC-131 cells exhibited higher expression of Cyclin D1 and E-Cadherin (Figure 3A) and CD44 (Figure 3B) compared to UPCI-SCC-154 cells. Furthermore, HPV-positive OSCC cells showed the upregulation of p16 (Figure 3C), a surrogate marker of HPV infection in oropharyngeal cancer 16-17.

### Wnt/βCatenin pathway is down-regulated in HPV-positive OSCC cells

**Figure 4:**
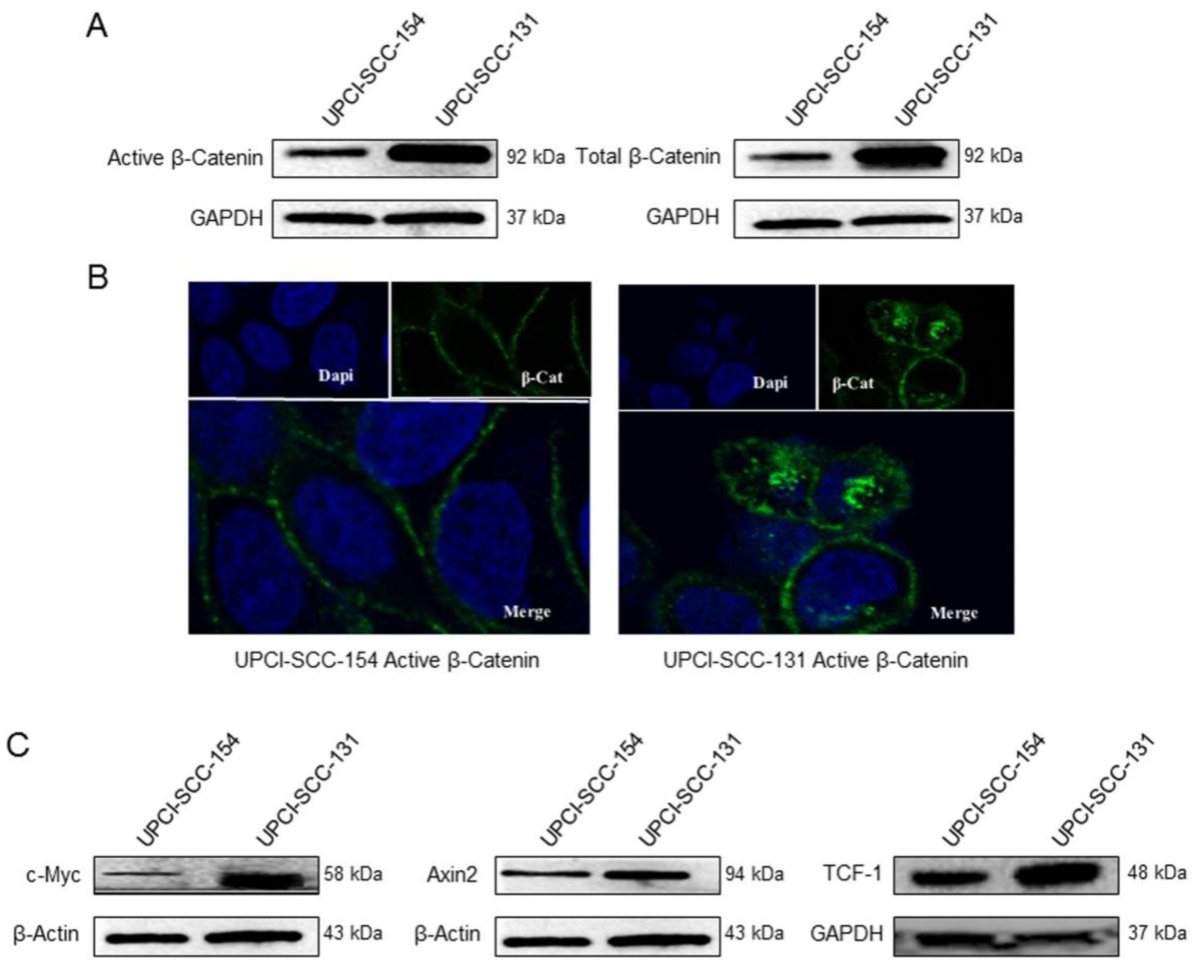
** Wnt/βCatenin signaling pathway analysis in UPCI-SCC-131 and UPCI-SCC-154 cells. (A)** Immunoblot analysis of total and active βCatenin protein levels in UPCI-SCC-131 and UPCI-SCC-154 cell lines. **(B)** Confocal microscopy analysis of active βCatenin intracellular distribution in UPCI-SCC-131 and UPCI-SCC-154 cell lines. Images were obtained with an original magnification of 60X. **(C)** Immunoblot analysis of cMyc, Axin-2 and Tcf-1 in UPCI-SCC-131 and UPCI-SCC-154 cell lines.

βCatenin, a subunit of the cadherin protein complex, is an intracellular signal transducer in Wnt signaling pathway, being able to translocate to the nucleus and induce the transcription of several target genes 18. The cellular level of βCatenin is mostly controlled by its phosphorylation, which drives the ubiquitination and the proteasomal degradation of the protein; whereas the activation of Wnt pathway results in increase of non-phosphorylated-βCatenin active form, thus enabling its transcriptional activity 19. Thus, to further study the differential activity of Wnt/βCatenin pathway between HPV-positive/negative OSCC cell lines, the expression of βCatenin and its non-phosphorylated active form was evaluated in both cell lines by immunoblot analysis. Indeed, HPV-negative UPCI-SCC-131 cells showed higher levels of total βCatenin and its active form compared to HPV-positive UPCI-SCC-154 (Figure 4A). In addition, confocal microscopy analysis showed higher nuclear accumulation of active βCatenin in UPCI-SCC-131 cells compared to UPCI-SCC-154 cells (Figure 4B), consistently with a lower activation of Wnt/βCatenin pathway in the HPV-positive cell line. Finally, UPCI-SCC-131 cells showed higher levels of *Axin-2, c-Myc* and *Tcf-1,* three Wnt/βCatenin target genes 20 (Figure 4C). Altogether, these data suggest that Wnt/βCatenin signaling pathway is positively modulated in our HPV-negative OSCC cell line compared to the HPV-positive counterpart.

### Higher activity of Wnt/βCatenin pathway correlates with stem cell traits

**Figure 5:**
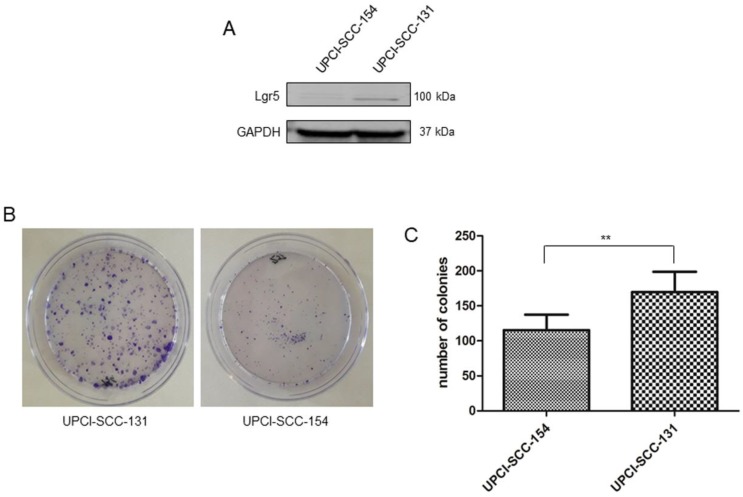
** Stem-like traits in UPCI-SCC-131 and UPCI-SCC-154 cell lines. (A-B)** Immunoblot analysis of Lgr5 protein expression **(A)** and representative images of colony forming assay **(B)** in UPCI-SCC-131 and UPCI-SCC-154 cell lines. **(C)** Colony forming assay quantification in UPCI-SCC-131 and UPCI-SCC-154 cell lines. **p value<0.05 compared to UPCI-SCC-131 cells.

Since Wnt/βCatenin pathway is responsible for cancer stem cell maintenance and self-renewal 21-23, the correlation between modulation of Wnt/βCatenin pathway and stem-like features was further evaluated in our panel of OSCC cells lines. Consistently with the higher level of CD44, an established marker of stemness in OSCCs 24 (Figure 3B), UPCI-SCC-131 cells exhibited higher expression of the cancer stem cell marker, Lgr5 25-26 compared to HPV-positive UPCI-SCC-154 cells (Figure 5A). In addition, colony forming assay showed higher number of colonies and increase of their size in UPCI-SCC-131 cells compared to UPCI-SCC-154 cells (Figure 5B-C). These results suggest that higher levels of activation of Wnt/βCatenin pathway correlate with more prominent stem-like traits in HPV-negative OSCC cells.

### miRNAlet-7e is involved in suppression of Wnt/βCatenin pathway in HPV-positive OSCC

**Figure 6:**
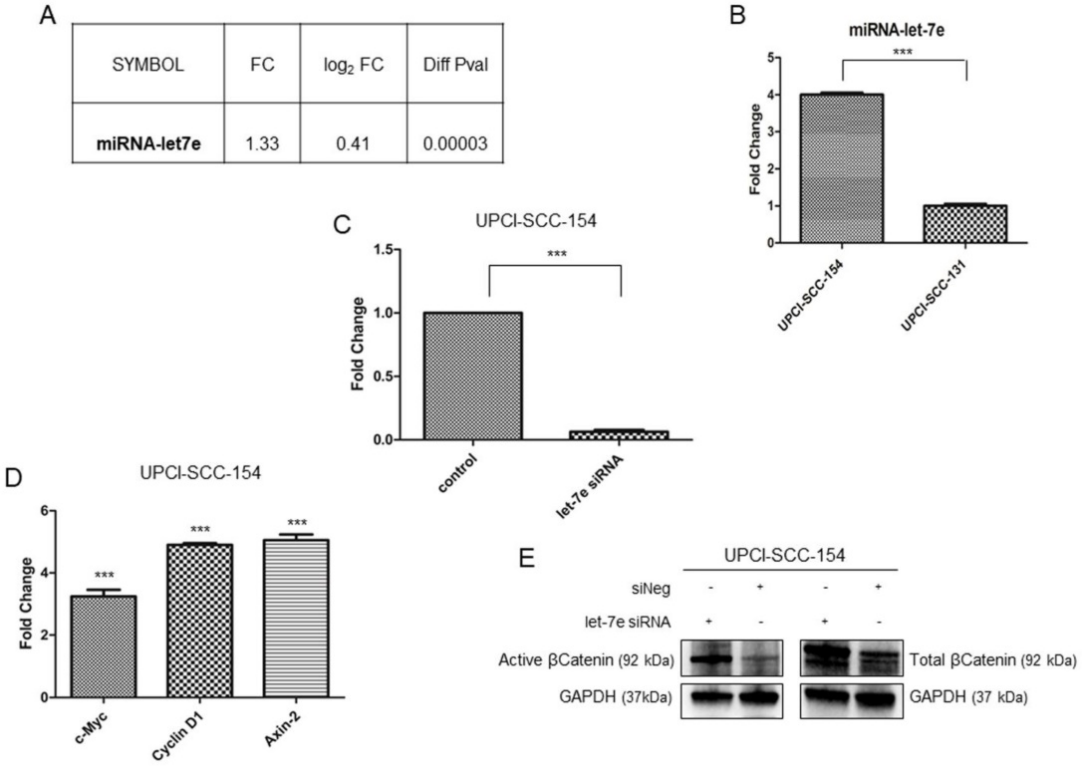
** Role of miRNAlet-7e in suppression of Wnt/βCatenin pathway in HPV-positive OSCC UPCI-SCC-154 cells. (A-B).** miRLet-7e expression in UPCI-SCC-154 compared to UPCI-SCC-131 cells, as obtained by gene expression **(A)** and real time PCR analysis (**B**) ***p value<0.0001 compared to UPCI-SCC-131 cells. **(C-D)** Expression of miRLet-7e **(C)** and *c-Myc, Axin2* and* Cyclin D1* genes **(D)** in UPCI-SCC-154 cells after transfection with control or miRNA Let-7e siRNA. ***p value<0.0001 compared to control**. (E)** Total and active βCatenin protein expression in UPCI-SCC-154 cells after transfection with control or Let-7e siRNA.

Microarray data suggest that multiple genes belonging to Wnt/βCatenin pathway are modulated in HPV-positive OSCC cells; thus, IPA software was used to identify potential upstream regulators responsible for modulation of Wnt/βCatenin signaling genes. Based on the list of Wnt/βCatenin pathway components or target genes modulated in our data set, IPA identified miRNA let-7e as potential upstream regulator. Indeed, miRNAlet-7e is known to act as a tumor suppressor by inhibiting the expression of βCatenin and stemness genes 27 and, thereby, is downregulated in oropharyngeal squamous carcinomas 28. The analysis of our data set (Figure 6A) and the subsequent real time validation assay (Figure 6B) showed higher levels of miRNAlet-7e in UPCI-SCC-154 compared to UPCI-SCC-131 cells. Thus, to prove the causative role of miRNA let-7e in suppressing Wnt/βCatenin in UPCI-SCC-154 HPV-positive cells, the expression of total βCatenin and its active form as well as three Wnt/βCatenin-target genes (i.e., *Axin-2, c-Myc* and *Cyclin D1*) were evaluated upon silencing of miRNA let-7e in UPCI-SCC-154 cells. Of note, miRNA let-7e silencing (Figure 6C) resulted in increased levels of total and active βCatenin (Figure 6E) and the parallel upregulation of *Axin-2, c-Myc* and *Cyclin D1* (Figure 6D), this suggesting that miRNAlet-7e is involved in suppression of Wnt/βCatenin pathway in HPV-positive OSCC cells.

### Wnt/βCatenin pathway is more active in HPV-negative HNSCC cell lines and tumors in two gene expression public datasets

The higher activation of Wnt/βCatenin pathway in HPV-negative versus HPV-positive HNSCCs was further investigated in two public datasets of HNSCC cell lines and tumors comparing the activation of Wnt/βCatenin pathway in HPV- positive versus HPV- negative tumors/cell lines both derived from patients with a smoking history. Two independent datasets were analyzed: i) GSE65858, which is a large dataset reporting gene expression data from 270 human HNSCCs and which allowed the comparison between 42 HPV16-positive versus 170 HPV-negative tumors, ii) GSE52088, which included gene expression data from 39 HNSCC cell lines and allowed the comparison between 2 HPV16 positive (i.e., SCC47 and SCC90 cells) and 11 HPV-negative (i.e., BIRC22, BIRC31, CAL27, DOK, H103, H357, H400, HN6, MSK921, OECM1 and SCC68 cells) cell lines. In both cases, only tumors/cell lines derived from patients with a smoking habit were analyzed, being the other cell lines/tumors uncertain for this issue. Clinicopathological and molecular characteristics of tumors from GSE65858 dataset and cell lines from the GSE52088 dataset are reported in, respectively, Supplementary Table S2 and Supplementary Table S3. The analysis of the GSE65858 dataset identified 482 differentially expressed genes (191 up- and 291 down-regulated) with -0.26>logFC>0.26, whereas the analysis of the GSE52088 dataset identified 1780 differentially-expressed genes (1091 up- and 689 down-regulated) with -0.26>logFC>0.26 (Supplementary Table S4). Interestingly, 16 up- and 36 down-regulated genes were coherently differentially expressed in both in house cell lines and HPV-positive/negative patients from GSE65858 dataset, as shown by the Venn diagram (Supplementary Figure S1 A-B). In addition, 23 up- and 26 down-regulated genes were coherently differentially expressed in both in house cell lines and HPV-positive/negative cell lines from GSE52088 dataset (Supplementary Figure S1 C-D).

Interestingly, the pathway analysis of gene expression data from both datasets identified the related Wnt/βCatenin gene set (Labbe WNT3a Targets) 29 as activated in HPV-negative tumors/cell lines (Table 2). More specifically, the analysis of gene expression data from HNSCC tumors identified 12/59 Wnt/βCatenin target genes significantly and coherently upregulated in HPV-negative tumors (adj. p-value 0.0036). By contrast, the analysis of HNSCC cell lines identified 9/110 Wnt/βCatenin target genes coherently downregulated in HPV-negative cell lines with a not-significant adjusted p-value. However, this difference was borderline significant (p-value 0.042) with a more relaxed threshold (Table 2). Altogether, these data provide a wider validation of our observation that Wnt/βCatenin pathway is more active in HPV-negative compared to HPV-positive HNSCC cells derived from smoking patients.

## Discussion

Patients bearing HPV-positive OSCCs are predominantly younger, non-smokers and non-drinkers and with a better prognosis compared to HPV-negative OSCC patients, even though the basis for these clinical differences are still largely unknown. In such a context, clinical evidences showed that the smoking habit might modify the more favorable clinical behavior of HPV-positive OSCCs 4. Indeed, several authors reported significant differences in survival rates between smoking and non-smoking HPV-positive OSCC patients, the latter being characterized by better outcome 5,30. In such a context, Ang et al. grouped patients with oropharyngeal tumors into three subgroups according to their prognosis: HPV-positive non-smoking patients with a longer survival (low risk tumors), HPV-negative smoking patients with the worst survival (high risk tumors) and HPV-positive and smoking patients with an intermediate outcome (intermediate risk tumors) 5. Therefore, patients with HPV-positivity and smoking habit may represent a distinct subgroup of oropharyngeal tumors, with a clinical phenotype that does not recapitulate classical HNSCCs positive for HPV infection. In such a perspective, a better understanding of the molecular pathobiology of this specific subgroup of oropharyngeal cancers is required.

This study addressed the relevance of HPV infection in the molecular profile of an oral squamous carcinoma cell line obtained from a smoking and drinking individual. Our data suggest that HPV-positive OSCC cells display a growth rate and a sensitivity to cisplatin comparable to HPV-negative cells. The gene expression analysis detected a differential activation of Wnt/βCatenin signaling pathway between the two cell lines, as shown by lower level of βCatenin and Wnt-target/related genes in HPV-positive compared to HPV-negative cells. Consistently, HPV-negative cells showed higher expression of stem cell antigens and increased clonogenic potential.

A major issue raised by these observations is their significance in the general context of the current literature about HPV-related oropharyngeal cancers. Indeed, the majority, but not all authors claim that Wnt/βCatenin pathway is up-regulated in patients with HPV-positive tumors 31-32. More specifically, *in vivo* studies showed a synergy between HPV infection and activation of Wnt/βCatenin signaling cascade 33-34, as well as Felthaus et al. suggested a specific influence of Wnt/βCatenin signaling on CSC proliferation in HPV-positive tumors 35. Thus, our observation that HPV-positive oral cancer cells are characterized by lower activation of Wnt/βCatenin signaling is apparently in contrast with the current view of the literature. However, it is important to note that the vast majority of studies that addressed the relationship between HPV infection, Wnt/βCatenin signaling modulation and stemness did not evaluated the relevance of the smoking habit in this process. Consistently with this interpretation, comparative gene expression analyses between HPV-positive and negative human oropharyngeal carcinomas, which did not consider the influence of other risk factors as the smoking habit, identified cell cycle regulation, DNA repair, apoptosis, DNA replication and recombination, but not Wnt/βCatenin signaling cascade, as major functions/pathways differentially active between HPV-positive and negative carcinomas 8-9. Thus, our study, to our knowledge, is the first that specifically evaluated the relevance of HPV infection in the context of oral carcinoma cell lines derived from smoking individuals and that identified Wnt/βCatenin signaling as differentially active in such a specific cell context. It is important to highlight that a limitation of our study is the head-to-head comparison between only two tobacco-related cell lines differing for the HPV status and the lack of data from human samples. Thus, to support our conclusions we extended our analysis to two independent public datasets reporting comparative gene expression data from human HNSCC tumors and cell lines. This analysis validated and reinforced the hypothesis that Wnt/βCatenin pathway is more active in HPV-negative versus HPV-positive tumors derived from patients with a smoking history, further supporting the concept that HPV-positive oropharyngeal carcinomas from a smoking individual are likely characterized by a different molecular profile compared to classical HPV-positive oropharyngeal cancer cells.

Recent studies highlighted the relevance of Wnt/βCatenin pathway in tumorigenesis, metastatic process, epithelial-to-mesenchymal transition (EMT) and regulation of cancer stem cell (CSC) expansion and maintenance 36. In fact, Wnt/βCatenin signaling cascade, among several other pathways, is a major driver in the intricate processes that govern CSC self-renewal 37-38 and EMT 39, this suggesting that the dysregulation of this pathway leads to cancer development 40. In such a context, the abnormal activation of Wnt/βCatenin canonical pathway has been associated with an increase in CSC proliferation and self-renewal in HNSCC 41-43. Thus, the correlation between higher activation of Wnt/βCatenin pathway and the expression of stem cell markers in our HPV-negative oral carcinoma cell line is consistent with the role of Wnt/βCatenin signaling in stemness maintenance in HNSCCs and other human malignancies 44. Furthermore, it has been proposed that both HPV infection and the smoking habit enhance the transition toward a mesenchymal phenotype in HNSCC cells 45-46. Indeed, HPV infection induces the repression of E-Cadherin and promotes EMT, this suggesting that HPV-related HNSCCs are likely to be characterized by a mesenchymal phenotype 47. However, this hypothesis is apparently in contrast with our observation that HPV-positive UPCI-SCC-154 cells, besides being characterized by lower expression of E-Cadherin compared to the HPV-negative UPCI-SCC-131 cells, are also characterized by lower levels of N-Cadherin, Vimentin and Twist-1, all known biomarkers of epithelial phenotype 48. This observation is consistent with a recent report suggesting that the association between HPV status and EMT in not statistically significant in a large cohort of 296 oropharyngeal carcinomas and this may be explained by the presence of mixed populations of HPV-positive patients with other associated risk factors (alcohol, tobacco) 49. Thus, based on all this evidence and consistently with the observation that Wnt/βCatenin pathway is also less active in these tumors/cell lines, we can hypothesize that HPV-positive HNSCC cells derived from smoking patients behave differently from canonical HPV-positive tumors.

The observation that Wnt/βCatenin pathway may be relevant in oropharyngeal tumorigenesis provides preclinical implications for development of new therapeutic strategies in these human malignancies. Indeed, the treatment with Wnt-C59, an inhibitor of Wnt/βCatenin signaling, showed inhibitory activity toward CSCs in nasopharyngeal carcinoma cells 50. Consistently, many studies reported reduced expression of βCatenin and suppression of CSC proliferation in HNSCC upon treatment with inhibitors of the Wnt/βCatenin canonical pathway, including secreted FRP4 (frizzled-related protein), ATRA (all-trans-retionic acid) and honokiol, an active natural compound 51-53. Thus, future studies are needed to identify subgroups of HNSCCs with constitutive activation of Wnt/βCatenin signaling and amenable for selective targeting of CSCs through inhibition of Wnt/βCatenin pathway. This hypothesis is relevant taking into account that current treatments for locally advanced and metastatic HNSCC patients rely of radiotherapy and chemotherapy and both treatments are known to kill proliferating cancer cells and not CSCs. Thus, the identification and validation of novel molecular targets to inhibit specifically CSCs are relevant clinical needs in this deadly disease.

Finally, our analysis identified miRNAlet-7e as potential upstream regulator of Wnt/βCatenin pathway modulation in our cell lines. Indeed, miRNA let-7e is known to be functionally involved in regulating the differentiation of stem cells/progenitors and reprogramming cell fates 54-56, and modulating βCatenin expression and Wnt pathway 27. Consistently with our observation, Vojtechova et al*.* reported that miRNA let-7e is down-regulated in HPV-negative HNSCC tumors 57. Thus, our data support the hypothesis that the differential activation of Wnt/βCatenin signaling between our cell lines may be related to the differential expression of miRNA let7e and that the expression of miRNA let7e may represent a tool to select oropharyngeal tumors with activation of Wnt/βCatenin pathway and stem-like traits.

## Conclusions

In conclusion, this is, to our knowledge, the first study that addressed, at molecular level, the significance of HPV infection in tobacco-related oropharyngeal cancer cell lines and showed that HPV-positive oral carcinoma cells from smoking patients are likely characterized by low activity of Wnt/βCatenin pathway compared to the HPV-negative counterpart. This unexpected molecular profile suggests that tobacco-associated HPV-positive OSCCs may represent a specific pathological entity with a molecular profile intermediate between classical HPV-positive and HPV-negative oropharyngeal cancers. Future studies are needed to confirm and validate this observation in selected subgroups of human oropharyngeal carcinomas and verify whether these molecular differences may explain the different outcome of tobacco-related HPV-positive tumors.

## Supplementary Material

Supplementary figures and tables.

## Figures and Tables

**Table 1:** Clinicopathological features of cell lines

Cell Line	UPCI-SCC-154	UPCI-SCC-131
**Gender**	M	M
**Age at diagnosis (years)**	54	73
**Ethnicity**	Caucasian	Caucasian
**Smoking**	Yes	Yes
**Alcohol**	Yes	Yes
**Origin**	n.p.	n.p.
**Primary tumor size**	Tongue	FOM
**Grading**	3	1
**TNM**	T4N2	T2N2
**TP53**	wt	wt
**11q13 amplification**	No	Yes
**p16**	Positive	Negative
**HPV+**	Positive	Negative

M, male; n.p., new primary; FOM, floor of mouth; wt, wild type

**Table 2:** Enrichment analysis of Wnt/βCatenin related pathway.

Gene Set	Data Set	Gene Ratio	p-value	Adj. p-value	Gene ID
Labbe Wnt3a Targets_Up	Tumors GSE65858	12/59	0.00010	0.0036	*Serpine1, Cav1, Ccnd1, Fabp5, Cyr61, Ctgf, Grem1, Cxcl1, Ahr, Ifit3, Nrp1, Ctsc*
Labbe Wnt3a Targets_Down	Cell Lines GSE52088	9/110	0.042	0.77	*Cdh11, Prkcb, Gng4, Tgm3, Tnfrsf1, Berbb3, Hpgd, Slc27a2, Pter*

Wnt/βCatenin related pathway enrichment in HPV-negative gene expression data from HNSCC tumors/cell lines derived from smoking patients (from GSE65858 and GSE52088 datasets)

## Abbreviations

BSA: bovine serum albumin
cDNA: complementary DNA
cRNA: complementary RNA
CSC: Cancer Stem Cells
DAPI: 4',6-diamidino-2-phenylindole
DEGs: differently expressed genes
DiffScore: differential score
FBS: Fetal bovine serum
*HNSCC*: Head and neck squamous cell carcinoma
*HPV*: Human Papilloma Virus
IPA: *Ingenuity Pathway Analysis*
MTT: 3-(4,5-Dimethylthiazol-2-yl)-2,5-Diphenyltetrazolium Bromide
OSCCs: oral squamous cell carcinomas
PBS: phosphate buffered saline
RT-PCR: real-time PCR
