# Supplementary material for: Comparative Gene Expression Profiling of Tobacco-Associated HPV-Positive versus Negative Oral Squamous Carcinoma Cell Lines
Source: Int J Med Sci. 2020 Jan 1;17(1):112–24. doi: 10.7150/ijms.35133 (PMC6945558; doi:10.7150/ijms.35133)
Supplement: Supplementary file 1 — Supplementary figures and tables. [file ijmsv17p0112s1.zip › Supplementary figure S1.pdf]

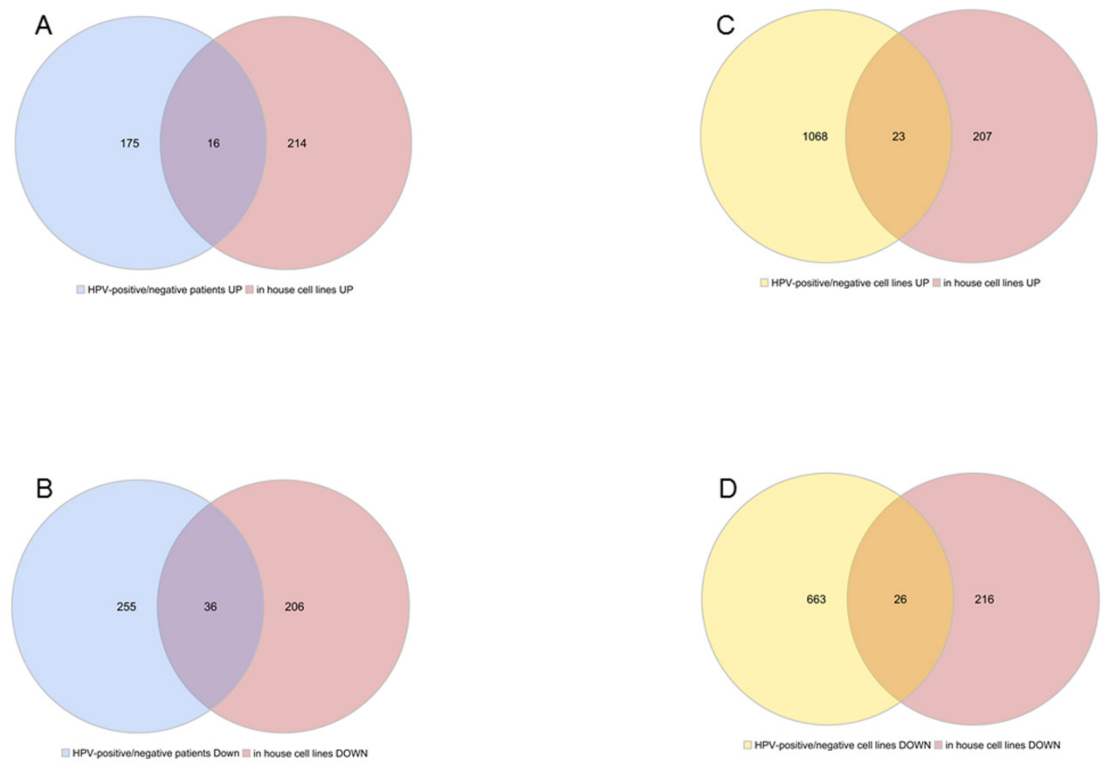

Supplementary Figure S1. Venn diagrams reporting up- (**A** and **C**) and down-regulated (**B** and **D**) genes coherently differentially expressed in both in house cell lines and HPV-positive/negative patients from GSE65858 dataset (**A-B**) and in both in house cell lines and HPV-positive/negative cell lines from GSE52088 dataset (**C-D**).
