# Supplementary material for: Comparative Gene Expression Profiling of Tobacco-Associated HPV-Positive versus Negative Oral Squamous Carcinoma Cell Lines
Source: Int J Med Sci. 2020 Jan 1;17(1):112–24. doi: 10.7150/ijms.35133 (PMC6945558; doi:10.7150/ijms.35133)
Supplement: Supplementary file 1 — Supplementary figures and tables. [file ijmsv17p0112s1.zip › Supplementary Table S1.docx]

**Supplementary Table S1.** Genes differently expressed in UPCI-SCC-154 compared to UPCI-SCC-131. Genes were selected with differential score cut-off set a ±18 (p<0.01)

| **ENTREZ**  **GENE-ID** | **Symbol** | **Expr Log ratio** | **Entrez Gene Name** |
| --- | --- | --- | --- |
| 760 | CA2 | 4.026 | carbonic anhydrase 2 |
| 7345 | UCHL1 | 3.774 | ubiquitin C-terminal hydrolase L1 |
| 3397 | ID1 | 3.529 | inhibitor of DNA binding 1, HLH protein |
| 100129681 | LOC100129681 | 3.095 | PREDICTED: Homo sapiens similar to NPC-A-7 (LOC100129681), mRNA. |
| 339766 | LOC339766 | 3.008 | PREDICTED: Homo sapiens hypothetical protein LOC339766 (LOC339766), mRNA. |
| 4312 | MMP1 | 2.981 | matrix metallopeptidase 1 |
| 119391 | GSTO2 | 2.953 | glutathione S-transferase omega 2 |
| 1029 | CDKN2A | 2.795 | cyclin dependent kinase inhibitor 2A |
| 6286 | S100P | 2.790 | S100 calcium binding protein P |
| 684 | BST2 | 2.742 | bone marrow stromal cell antigen 2 |
| 5918 | RARRES1 | 2.702 | retinoic acid receptor responder 1 |
| 94234 | FOXQ1 | 2.695 | forkhead box Q1 |
| 10170 | DHRS9 | 2.642 | dehydrogenase/reductase 9 |
| 346887 | LOC346887 | 2.471 | PREDICTED: Homo sapiens similar to solute carrier family 16 (monocarboxylic acid transporters), member 14 (LOC346887), mRNA. |
| 79083 | MLPH | 2.448 | melanophilin |
| 85016 | C11orf70 | 2.431 | chromosome 11 open reading frame 70 |
| 3489 | IGFBP6 | 2.411 | insulin like growth factor binding protein 6 |
| 283212 | KLHL35 | 2.399 | kelch like family member 35 |
| 9536 | PTGES | 2.393 | prostaglandin E synthase |
| 4128 | MAOA | 2.391 | monoamine oxidase A |
| 56603 | CYP26B1 | 2.375 | cytochrome P450 family 26 subfamily B member 1 |
| 2946 | GSTM2 | 2.252 | glutathione S-transferase mu 2 |
| 11199 | ANXA10 | 2.239 | annexin A10 |
| 51191 | HERC5 | 2.237 | HECT and RLD domain containing E3 ubiquitin protein ligase 5 |
| 445328 | ARHGEF5L | 2.231 | Rho guanine nucleotide exchange factor 35 |
| 26022 | TMEM98 | 2.223 | transmembrane protein 98 |
| 10413 | YAP1 | 2.212 | Yes associated protein 1 |
| 5673 | PSG5 | 2.147 | pregnancy specific beta-1-glycoprotein 5 |
| 9518 | GDF15 | 2.116 | growth differentiation factor 15 |
| 84259 | DCUN1D5 | 2.073 | defective in cullin neddylation 1 domain containing 5 |
| 2644 | GCHFR | 2.073 | GTP cyclohydrolase I feedback regulator |
| 5801 | PTPRR | 2.069 | protein tyrosine phosphatase, receptor type R |
| 3169 | FOXA1 | 2.062 | forkhead box A1 |
| 5678 | PSG9 | 2.055 | pregnancy specific beta-1-glycoprotein 9 |
| 220 | ALDH1A3 | 2.019 | aldehyde dehydrogenase 1 family member A3 |
| 6920 | TCEA3 | 2.010 | transcription elongation factor A3 |
| 649841 | LOC649841 | 1.994 | PREDICTED: Homo sapiens similar to protein immuno-reactive with anti-PTH polyclonal antibodies (LOC649841), mRNA. |
| 2944 | GSTM1 | 1.982 | glutathione S-transferase mu 1 |
| 652995 | UCA1 | 1.967 | urothelial cancer associated 1 (non-protein coding) |
| 11166 | SOX21 | 1.948 | SRY-box 21 |
| 133 | ADM | 1.948 | adrenomedullin |
| 10683 | DLL3 | 1.931 | delta like canonical Notch ligand 3 |
| 1514 | CTSL1 | 1.910 | cathepsin L |
| 388494 | LOC388494 | 1.906 | PREDICTED: Homo sapiens hypothetical gene supported by AL365406; BC034005 (LOC388494), mRNA. |
| 284023 | LOC284023 | 1.900 | PREDICTED: Homo sapiens hypothetical protein LOC284023, transcript variant 3 (LOC284023), mRNA. |
| 329 | BIRC2 | 1.889 | baculoviral IAP repeat containing 2 |
| 131474 | CHCHD4 | 1.885 | coiled-coil-helix-coiled-coil-helix domain containing 4 |
| 55930 | MYO5C | 1.880 | myosin VC |
| 3399 | ID3 | 1.862 | inhibitor of DNA binding 3, HLH protein |
| 26064 | RAI14 | 1.855 | retinoic acid induced 14 |
| 649853 | HLA-A29.1 | 1.844 | major histo compatibility complex class I HLA A-29.1 |
| 9500 | MAGED1 | 1.842 | MAGE family member D1 |
| 22943 | DKK1 | 1.833 | dickkopf WNT signaling pathway inhibitor 1 |
| 3006 | HIST1H1C | 1.815 | histone cluster 1 H1 family member c |
| 79659 | DYNC2H1 | 1.756 | dynein cytoplasmic 2 heavy chain 1 |
| 970 | CD70 | 1.755 | CD70 molecule |
| 54460 | MRPS21 | 1.752 | mitochondrial ribosomal protein S21 |
| 58495 | OVOL2 | 1.747 | ovo like zinc finger 2 |
| 6638 | SNRPN | 1.738 | small nuclear ribonucleoprotein polypeptide N |
| 19 | ABCA1 | 1.737 | ATP binding cassette subfamily A member 1 |
| 5646 | PRSS3 | 1.728 | protease, serine 3 |
| 8926 | SNURF | 1.719 | SNRPN upstream reading frame |
| 23645 | PPP1R15A | 1.708 | protein phosphatase 1 regulatory subunit 15A |
| 155066 | ATP6V0E2 | 1.701 | ATPase H+ transporting V0 subunit e2 |
| 9920 | KBTBD11 | 1.701 | kelch repeat and BTB domain containing 11 |
| 3948 | LDHC | 1.701 | lactate dehydrogenase C |
| 23762 | OSBP2 | 1.678 | oxysterol binding protein 2 |
| 25894 | PLEKHG4 | 1.677 | pleckstrin homology and RhoGEF domain containing G4 |
| 729642 | LOC729642 | 1.673 | PREDICTED: Homo sapiens hypothetical LOC729642 (LOC729642), mRNA. |
| 3202 | HOXA5 | 1.672 | homeobox A5 |
| 23231 | SEL1L3 | 1.672 | SEL1L family member 3 |
| 7169 | TPM2 | 1.651 | tropomyosin 2 (beta) |
| 100129673 | LOC100129673 | 1.649 | PREDICTED: Homo sapiens similar to hCG2042915 (LOC100129673), mRNA. |
| 283932 | LOC283932 | 1.636 | Homo sapiens hypothetical protein LOC283932 (LOC283932), mRNA. |
| 8370 | HIST2H4A | 1.617 | histone cluster 2 H4 family member a |
| 9987 | HNRPDL | 1.598 | heterogeneous nuclear ribonucleoprotein D like |
| 3491 | CYR61 | 1.591 | cysteine rich angiogenic inducer 61 |
| 3398 | ID2 | 1.584 | inhibitor of DNA binding 2, HLH protein |
| 7508 | XPC | 1.579 | XPC complex subunit, DNA damage recognition and repair factor |
| 3636 | INPPL1 | 1.577 | inositol polyphosphate phosphatase like 1 |
| 5547 | PRCP | 1.575 | prolylcarboxypeptidase |
| 64145 | ZFYVE20 | 1.572 | rabenosyn, RAB effector |
| 3486 | IGFBP3 | 1.560 | insulin like growth factor binding protein 3 |
| 29903 | CCDC106 | 1.556 | coiled-coil domain containing 106 |
| 84992 | PIGY | 1.552 | phosphatidylinositol glycan anchor biosynthesis class Y |
| 27258 | LSM3 | 1.550 | LSM3 homolog, U6 small nuclear RNA and mRNA degradation associated |
| 1528 | CYB5A | 1.537 | cytochrome b5 type A |
| 114908 | TMEM123 | 1.533 | transmembrane protein 123 |
| 140465 | MYL6B | 1.530 | myosin light chain 6B |
| 223 | ALDH9A1 | 1.530 | aldehyde dehydrogenase 9 family member A1 |
| 4241 | MFI2 | 1.527 | melanotransferrin |
| 9123 | SLC16A3 | 1.526 | solute carrier family 16 member 3 |
| 3620 | IDO1 | 1.526 | indoleamine 2,3-dioxygenase 1 |
| 214 | ALCAM | 1.515 | activated leukocyte cell adhesion molecule |
| 647302 | LOC647302 | 1.511 | PREDICTED: Homo sapiens misc_RNA (LOC647302), miscRNA. |
| 112398 | EGLN2 | 1.506 | egl-9 family hypoxia inducible factor 2 |
| 4240 | MFGE8 | 1.502 | milk fat globule-EGF factor 8 protein |
| 11098 | PRSS23 | 1.501 | protease, serine 23 |
| 6303 | SAT1 | 1.500 | spermidine/spermine N1-acetyltransferase 1 |
| 3860 | KRT13 | 1.495 | keratin 13 |
| 730995 | LOC730995 | 1.495 | PREDICTED: Homo sapiens similar to ankyrin repeat domain 36 (LOC730995), mRNA. |
| 60370 | AVPI1 | 1.481 | arginine vasopressin induced 1 |
| 7076 | TIMP1 | 1.476 | TIMP metallopeptidase inhibitor 1 |
| 1649 | DDIT3 | 1.470 | DNA damage inducible transcript 3 |
| 196792 | FAM24B | 1.454 | family with sequence similarity 24 member B |
| 8495 | PPFIBP2 | 1.449 | PPFIA binding protein 2 |
| 51665 | ASB1 | 1.449 | ankyrin repeat and SOCS box containin |
| 55638 | GOLSYN | 1.447 | Golgi-localited syntaphilin-relate protein |
| 27148 | STK36 | 1.442 | serine/threonine kinase 36 |
| 90835 | C16orf93 | 1.440 | coiled-coil domain containing 189 |
| 56925 | LXN | 1.431 | latexin |
| 8876 | VNN1 | 1.427 | vanin 1 |
| 113802 | C1orf59 | 1.427 | HEN1 methyltransferase homolog 1 |
| 79191 | IRX3 | 1.395 | iroquois homeobox 3 |
| 64332 | NFKBIZ | 1.388 | NFKB inhibitor zeta |
| 2040 | STOM | 1.378 | stomatin |
| 29950 | SERTAD1 | 1.374 | SERTA domain containing 1 |
| 23225 | NUP210 | 1.372 | nucleoporin 210 |
| 163126 | EID2 | 1.370 | EP300 interacting inhibitor of differentiation 2 |
| 25978 | CHMP2B | 1.366 | charged multivesicular body protein 2B |
| 3620 | INDO | 1.361 | indoleamine-pyrrole 2,3 dioxygenase (INDO) |
| 306 | ANXA3 | 1.359 | annexin A3 |
| 6907 | TBL1X | 1.356 | transducin beta like 1X-linked |
| 100133662 | LOC100133662 | 1.355 | PREDICTED: Homo sapiens hypothetical protein LOC100133662 (LOC100133662), mRNA. |
| 3306 | HSPA2 | 1.354 | heat shock protein family A (Hsp70) member 2 |
| 56650 | CLDND1 | 1.353 | claudin domain containing 1 |
| 50805 | IRX4 | 1.343 | iroquois homeobox 4 |
| 240 | ALOX5 | 1.341 | arachidonate 5-lipoxygenase |
| 4696 | NDUFA3 | 1.340 | NADH:ubiquinone oxidoreductase subunit A3 |
| 400986 | LOC400986 | 1.329 | PREDICTED: Homo sapiens protein immuno-reactive with anti-PTH polyclonal antibodies (LOC400986), mRNA. |
| 112616 | CMTM7 | 1.323 | CKLF like MARVEL transmembrane domain containing 7 |
| 60492 | CCDC90B | 1.323 | coiled-coil domain containing 90B |
| 4599 | MX1 | 1.321 | MX dynamin like GTPase 1 |
| 51244 | C3orf19 | 1.321 | coiled-coil domain containing 174 |
| 339324 | ZNF260 | 1.316 | zinc finger protein 260 |
| 6192 | RPS4Y1 | 1.313 | ribosomal protein S4, Y-linked 1 |
| 8835 | SOCS2 | 1.312 | suppressor of cytokine signaling 2 |
| 56675 | NRIP3 | 1.311 | nuclear receptor interacting protein 3 |
| 134147 | CMBL | 1.311 | carboxymethylenebutenolidase homolog |
| 2920 | CXCL2 | 1.309 | C-X-C motif chemokine ligand 2 |
| 25945 | PVRL3 | 1.307 | nectin cell adhesion molecule 3 |
| 84034 | EMILIN2 | 1.292 | elastin microfibril interfacer 2 |
| 84215 | ZNF541 | 1.291 | zinc finger protein 541 |
| 7049 | TGFBR3 | 1.289 | transforming growth factor beta receptor 3 |
| 158158 | RASEF | 1.277 | RAS and EF-hand domain containing |
| 51316 | PLAC8 | 1.276 | placenta specific 8 |
| 10409 | BASP1 | 1.276 | brain abundant membrane attached signal protein 1 |
| 85459 | KIAA1731 | 1.273 | centrosomal protein 295 |
| 284370 | ZNF615 | 1.269 | zinc finger protein 615 |
| 83595 | SOX7 | 1.262 | SRY-box 7 |
| 284757 | LOC284757 | 1.262 | Homo sapiens hypothetical protein LOC284757 (LOC284757), mRNA. |
| 1843 | DUSP1 | 1.258 | dual specificity phosphatase 1 |
| 218 | ALDH3A1 | 1.258 | aldehyde dehydrogenase 3 family member A1 |
| 80336 | PABPC1L | 1.256 | poly(A) binding protein cytoplasmic 1 like |
| 3231 | HOXD1 | 1.252 | homeobox D1 |
| 27018 | NGFRAP1 | 1.232 | brain expressed X-linked 3 |
| 90313 | TP53I13 | 1.221 | tumor protein p53 inducible protein 13 |
| 593 | BCKDHA | 1.217 | branched chain keto acid dehydrogenase E1, alpha polypeptide |
| 54504 | CPVL | 1.211 | carboxypeptidase, vitellogenic like |
| 51466 | EVL | 1.211 | Enah/Vasp-like |
| 29785 | CYP2S1 | 1.210 | cytochrome P450 family 2 subfamily S member 1 |
| 644 | BLVRA | 1.208 | biliverdin reductase A |
| 688 | KLF5 | 1.202 | Kruppel like factor 5 |
| 401115 | LOC401115 | 1.200 | PREDICTED: Homo sapiens hypothetical gene supported by BC038466; BC062790 (LOC401115), mRNA. |
| 644907 | LOC644907 | 1.199 | Homo sapiens hCG18290 (LOC644907), mRNA. |
| 54470 | ARMCX6 | 1.199 | armadillo repeat containing, X-linked 6 |
| 152519 | NIPAL1 | 1.196 | NIPA like domain containing 1 |
| 6446 | SGK | 1.196 | serum/glucocorticoid regulated kinase 1 |
| 11145 | HRASLS3 | 1.194 | phospholipase A2 group XVI |
| 3934 | LCN2 | 1.193 | lipocalin 2 |
| 9823 | ARMCX2 | 1.191 | armadillo repeat containing, X-linked 2 |
| 8337 | HIST2H2AA3 | 1.189 | histone cluster 2 H2A family member a3 |
| 58487 | CREBZF | 1.187 | CREB/ATF bZIP transcription factor |
| 387066 | SNHG5 | 1.184 | small nucleolar RNA host gene 5 |
| 5894 | RAF1 | 1.182 | Raf-1 proto-oncogene, serine/threonine kinase |
| 330 | BIRC3 | 1.182 | baculoviral IAP repeat containing 3 |
| 10099 | TSPAN3 | 1.177 | tetraspanin 3 |
| 2217 | FCGRT | 1.177 | Fc fragment of IgG receptor and transporter |
| 27074 | LAMP3 | 1.176 | lysosomal associated membrane protein 3 |
| 5090 | PBX3 | 1.176 | PBX homeobox 3 |
| 54457 | TAF7L | 1.172 | TATA-box binding protein associated factor 7 like |
| 55502 | HES6 | 1.172 | hes family bHLH transcription factor 6 |
| 100130178 | LOC100130178 | 1.165 | PREDICTED: Homo sapiens misc_RNA (LOC100130178), miscRNA. |
| 5920 | RARRES3 | 1.161 | retinoic acid receptor responder 3 |
| 100008588 | LOC100008588 | 1.160 | Homo sapiens 18S ribosomal RNA (LOC100008588), non-coding RNA. |
| 51503 | CWC15 | 1.158 | CWC15 spliceosome associated protein homolog |
| 723790 | HIST2H2AA4 | 1.152 | histone cluster 2,H2aa4 |
| 5125 | PCSK5 | 1.150 | proprotein convertase subtilisin/kexin type 5 |
| 51309 | ARMCX1 | 1.147 | armadillo repeat containing, X-linked 1 |
| 79901 | CYBRD1 | 1.143 | cytochrome b reductase 1 |
| 2152 | F3 | 1.143 | coagulation factor III, tissue factor |
| 146771 | TCAM1 | 1.128 | testicular cell adhesion molecule 1, pseudogene |
| 731314 | LOC731314 | 1.127 | PREDICTED: Homo sapiens similar to H2A histone family, member X (LOC731314), mRNA. |
| 10063 | COX17 | 1.121 | COX17, cytochrome c oxidase copper chaperone |
| 5445 | PON2 | 1.117 | paraoxonase 2 |
| 116843 | C6orf192 | 1.117 | solute carrier family 18 member B1 |
| 55344 | PLCXD1 | 1.113 | phosphatidylinositol specific phospholipase C X domain containing 1 |
| 8644 | AKR1C3 | 1.109 | aldo-keto reductase family 1 member C3 |
| 55120 | FANCL | 1.106 | Fanconi anemia complementation group L |
| 8463 | TEAD2 | 1.105 | TEA domain transcription factor 2 |
| 84233 | TMEM126A | 1.103 | transmembrane protein 126A |
| 6446 | SGK1 | 1.102 | serum/glucocorticoid regulated kinase 1 |
| 79188 | TMEM43 | 1.101 | transmembrane protein 43 |
| 9647 | PPM1F | 1.100 | protein phosphatase, Mg2+/Mn2+ dependent 1F |
| 8301 | PICALM | 1.099 | phosphatidylinositol binding clathrin assembly protein |
| 4925 | NUCB2 | 1.098 | nucleobindin 2 |
| 56907 | SPIRE1 | 1.096 | spire type actin nucleation factor 1 |
| 5672 | PSG4 | 1.095 | pregnancy specific beta-1-glycoprotein 4 |
| 100133923 | LOC100133923 | 1.092 | PREDICTED: Homo sapiens similar to H2A histone family, member X (LOC731314), mRNA. |
| 283489 | ZNF828 | 1.092 | chromosome alignment maintaining phosphoprotein 1 |
| 23512 | SUZ12 | 1.089 | SUZ12 polycomb repressive complex 2 subunit |
| 8334 | HIST1H2AC | 1.087 | histone cluster 1 H2A family member c |
| 51138 | COPS4 | 1.087 | COP9 signalosome subunit 4 |
| 1434 | CSE1L | 1.078 | chromosome segregation 1 like |
| 51501 | C11orf73 | 1.076 | Hikeshi, heat shock protein nuclear import factor |
| 7001 | PRDX2 | 1.075 | peroxiredoxin 2 |
| 4091 | SMAD6 | 1.071 | SMAD family member 6 |
| 219738 | C10orf35 | 1.070 | chromosome 10 open reading frame 35 |
| 647349 | LOC647349 | 1.066 | PREDICTED: Homo sapiens similar to AP-3 complex subunit sigma-1 (Adapter-related protein complex 3 sigma-1 subunit) (Sigma-adaptin 3a) (AP-3 complex sigma-3A subunit) (Sigma-3A-adaptin) (LOC647349), mRNA. |
| 2790 | GNG10 | 1.064 | G protein subunit gamma 10 |
| 51192 | CKLF | 1.063 | chemokine like factor |
| 2619 | GAS1 | 1.063 | growth arrest specific 1 |
| 3460 | IFNGR2 | 1.061 | interferon gamma receptor 2 |
| 55863 | TMEM126B | 1.057 | transmembrane protein 126B |
| 100132439 | LOC100132439 | 1.055 | PREDICTED: Homo sapiens similar to Protein FAM27E3 (LOC100132439), mRNA. |
| 10370 | CITED2 | 1.053 | Cbp/p300 interacting transactivator with Glu/Asp rich carboxy-terminal domain 2 |
| 1999 | ELF3 | 1.046 | E74 like ETS transcription factor 3 |
| 2308 | FOXO1 | 1.045 | forkhead box O1 |
| 132001 | C3orf31 | 1.044 | TAM41 mitochondrial translocator assembly and maintenance homolog |
| 6284 | S100A13 | 1.041 | S100 calcium binding protein A13 |
| 10780 | ZNF234 | 1.039 | zinc finger protein 234 |
| 7596 | ZNF45 | 1.038 | zinc finger protein 45 |
| 5675 | PSG6 | 1.038 | pregnancy specific beta-1-glycoprotein 6 |
| 55170 | PRMT6 | 1.038 | protein arginine methyltransferase 6 |
| 23164 | MPRIP | 1.037 | myosin phosphatase Rho interacting protein |
| 94121 | SYTL4 | 1.036 | synaptotagmin like 4 |
| 144348 | ZNF664 | 1.034 | zinc finger protein 664 |
| 28984 | C13orf15 | 1.032 | regulator of cell cycle |
| 2878 | GPX3 | 1.031 | glutathione peroxidase 3 |
| 154791 | C7orf55 | 1.029 | formation of mitochondrial complex V assembly factor 1 homolog |
| 54742 | LY6K | 1.029 | lymphocyte antigen 6 family member K |
| 339344 | MYPOP | 1.024 | Myb related transcription factor, partner of profilin |
| 645553 | LOC645553 | 1.021 | PREDICTED: Homo sapiens hypothetical LOC645553 (LOC645553), mRNA. |
| 7593 | MZF1 | 1.021 | myeloid zinc finger 1 |
| 950 | SCARB2 | 1.017 | scavenger receptor class B member 2 |
| 10981 | RAB32 | 1.016 | RAB32, member RAS oncogene family |
| 55020 | TTC38 | 1.015 | tetratricopeptide repeat domain 38 |
| 5866 | RAB3IL1 | 1.014 | RAB3A interacting protein like 1 |
| 55556 | ENOSF1 | 1.014 | enolase superfamily member 1 |
| 126299 | ZNF428 | 1.014 | zinc finger protein 428 |
| 286676 | ILDR1 | 1.012 | immunoglobulin like domain containing receptor 1 |
| 6484 | ST3GAL4 | 1.011 | ST3 beta-galactoside alpha-2,3-sialyltransferase 4 |
| 3184 | HNRNPD | 1.009 | heterogeneous nuclear ribonucleoprotein D |
| 85026 | C9orf37 | 1.008 | ARRDC1 antisense RNA 1 |
| 4798 | NFRKB | 1.007 | nuclear factor related to kappaB binding protein |
| 23609 | MKRN2 | 1.004 | makorin ring finger protein 2 |
| 128876 | FAM83C | -1.006 | family with sequence similarity 83 member C |
| 6892 | TAPBP | -1.008 | TAP binding protein |
| 27230 | SERP1 | -1.009 | stress associated endoplasmic reticulum protein 1 |
| 79744 | ZNF419 | -1.010 | zinc finger protein 419 |
| 126282 | TNFAIP8L1 | -1.011 | TNF alpha induced protein 8 like 1 |
| 6137 | RPL13 | -1.011 | ribosomal protein L13 |
| 256691 | MAMDC2 | -1.015 | MAM domain containing 2 |
| 7136 | TNNI2 | -1.018 | troponin I2, fast skeletal type |
| 79786 | KLHL36 | -1.018 | kelch like family member 36 |
| 60 | ACTB | -1.020 | actin beta |
| 645166 | LOC645166 | -1.024 | lymphocyte-specific protein 1 pseudogene |
| 130814 | PQLC3 | -1.025 | PQ loop repeat containing 3 |
| 3312 | HSPA8 | -1.025 | heat shock protein family A (Hsp70) member 8 |
| 50619 | DEF6 | -1.026 | DEF6, guanine nucleotide exchange factor |
| 729768 | LOC729768 | -1.026 | PREDICTED: Homo sapiens misc_RNA (LOC729768), miscRNA. |
| 11227 | GALNT5 | -1.027 | polypeptide N-acetylgalactosaminyltransferase 5 |
| 79763 | ISOC2 | -1.031 | isochorismatase domain containing 2 |
| 391075 | LOC391075 | -1.032 | PREDICTED: Homo sapiens misc_RNA (LOC391075), miscRNA. |
| 90850 | ZNF598 | -1.033 | zinc finger protein 598 |
| 79080 | CCDC86 | -1.035 | coiled-coil domain containing 86 |
| 4942 | OAT | -1.036 | ornithine aminotransferase |
| 114907 | FBXO32 | -1.040 | F-box protein 32 |
| 28234 | SLCO1B3 | -1.040 | solute carrier organic anion transporter family member 1B3 |
| 100132564 | LOC100132564 | -1.041 | PREDICTED: Homo sapiens hypothetical protein LOC100132564 (LOC100132564), mRNA. |
| 9525 | VPS4B | -1.043 | vacuolar protein sorting 4 homolog B |
| 441013 | LOC441013 | -1.045 | PREDICTED: Homo sapiens misc_RNA (LOC441013), miscRNA. |
| 3134 | HLA-F | -1.046 | major histocompatibility complex, class I, F |
| 29922 | NME7 | -1.048 | NME/NM23 family member 7 |
| 8710 | SERPINB7 | -1.048 | serpin family B member 7 |
| 3107 | HLA-C | -1.049 | major histocompatibility complex, class I, C |
| 2196 | FAT2 | -1.049 | FAT atypical cadherin 2 |
| 81853 | TMEM14B | -1.049 | transmembrane protein 14B |
| 10874 | NMU | -1.050 | neuromedin U |
| 730010 | BRI3P1 | -1.054 | brain protein I3 pseudogene 1 |
| 55552 | HSZFP36 | -1.055 | ZFP-36 for a zinc finger protein |
| 55076 | TMEM45A | -1.057 | transmembrane protein 45A |
| 55277 | FLJ10986 | -1.058 | hypothetical protein FLJ10986 |
| 9651 | PLCH2 | -1.059 | phospholipase C eta 2 |
| 11014 | KDELR2 | -1.059 | KDEL endoplasmic reticulum protein retention receptor 2 |
| 3105 | HLA-A | -1.061 | major histocompatibility complex, class I, A |
| 6288 | SAA1 | -1.065 | serum amyloid A1 |
| 440 | ASNS | -1.068 | asparagine synthetase (glutamine-hydrolyzing) |
| 728939 | PDZK1P1 | -1.068 | PDZ domain containing 1 pseudogene 1 |
| 4041 | LRP5 | -1.068 | LDL receptor related protein 5 |
| 6575 | SLC20A2 | -1.071 | solute carrier family 20 member 2 |
| 7923 | HSD17B8 | -1.071 | hydroxysteroid 17-beta dehydrogenase 8 |
| 147694 | ZNF548 | -1.072 | zinc finger protein 548 |
| 1033 | CDKN3 | -1.072 | cyclin dependent kinase inhibitor 3 |
| 9582 | APOBEC3B | -1.075 | apolipoprotein B mRNA editing enzyme catalytic subunit 3B |
| 55748 | CNDP2 | -1.075 | carnosine dipeptidase 2 |
| 3371 | TNC | -1.076 | tenascin C |
| 25921 | ZDHHC5 | -1.077 | zinc finger DHHC-type containing 5 |
| 644879 | LOC644879 | -1.080 | PREDICTED: Homo sapiens misc_RNA (LOC644879), miscRNA. |
| 2744 | GLS | -1.084 | glutaminase |
| 205 | AK3L1 | -1.086 | adenylate kinase 4 |
| 645173 | LOC645173 | -1.086 | PREDICTED: Homo sapiens misc_RNA (LOC645173), miscRNA. |
| 1825 | DSC3 | -1.087 | desmocollin 3 |
| 286444 | LOC286444 | -1.088 | PREDICTED: Homo sapiens misc_RNA (LOC286444), miscRNA. |
| 3217 | HOXB7 | -1.091 | homeobox B7 |
| 11164 | NUDT5 | -1.091 | nudix hydrolase 5 |
| 729679 | LOC729679 | -1.092 | PREDICTED: Homo sapiens misc_RNA (LOC729679), miscRNA. |
| 4494 | MT1F | -1.092 | metallothionein 1F |
| 2180 | ACSL1 | -1.092 | acyl-CoA synthetase long-chain family member 1 |
| 6310 | ATXN1 | -1.093 | ataxin 1 |
| 1605 | DAG1 | -1.094 | dystroglycan 1 |
| 402221 | LOC402221 | -1.094 | PREDICTED: Homo sapiens similar to actin alpha 1 skeletal muscle protein (LOC402221), mRNA. |
| 152137 | CCDC50 | -1.096 | coiled-coil domain containing 50 |
| 51693 | TRAPPC2L | -1.097 | trafficking protein particle complex 2 like |
| 794 | CALB2 | -1.097 | calbindin 2 |
| 1832 | DSP | -1.098 | desmoplakin |
| 6745 | SSR1 | -1.102 | signal sequence receptor subunit 1 |
| 1140 | CHRNB1 | -1.104 | cholinergic receptor nicotinic beta 1 subunit |
| 100133941 | CD24 | -1.104 | CD24 molecule |
| 5190 | PEX6 | -1.109 | peroxisomal biogenesis factor 6 |
| 7086 | TKT | -1.110 | transketolase |
| 4118 | MAL | -1.112 | mal, T-cell differentiation protein |
| 7965 | AIMP2 | -1.114 | aminoacyl tRNA synthetase complex interacting multifunctional protein 2 |
| 163351 | GBP6 | -1.116 | guanylate binding protein family member 6 |
| 928 | CD9 | -1.117 | CD9 molecule |
| 7280 | TUBB2A | -1.118 | tubulin beta 2A class IIa |
| 253782 | LASS6 | -1.119 | ceramide synthase 6 |
| 651397 | LOC651397 | -1.120 | PREDICTED: Homo sapiens misc_RNA (LOC651397), miscRNA. |
| 1265 | CNN2 | -1.120 | calponin 2 |
| 649970 | LOC649970 | -1.121 | PREDICTED: Homo sapiens similar to creatine kinase, mitochondrial 1B precursor (LOC649970), mRNA. |
| 25923 | ATL3 | -1.122 | atlastin GTPase 3 |
| 163782 | KANK4 | -1.125 | ankyrin repeat domain 38 |
| 7378 | UPP1 | -1.126 | uridine phosphorylase 1 |
| 84310 | C7orf50 | -1.127 | chromosome 7 open reading frame 50 |
| 3949 | LDLR | -1.137 | low density lipoprotein receptor |
| 114926 | C8orf40 | -1.137 | small integral membrane protein 19 |
| 406988 | MIR205 | -1.140 | microRNA 205 |
| 58477 | SRPRB | -1.140 | SRP receptor beta subunit |
| 899 | CCNF | -1.151 | cyclin F |
| 8815 | BANF1 | -1.153 | barrier to autointegration factor 1 |
| 51111 | SUV420H1 | -1.154 | lysine methyltransferase 5B |
| 402 | ARL2 | -1.155 | ADP ribosylation factor like GTPase 2 |
| 153769 | SH3RF2 | -1.156 | SH3 domain containing ring finger 2 |
| 1491 | CTH | -1.158 | cystathionine gamma-lyase |
| 729406 | LOC729406 | -1.159 | PREDICTED: Homo sapiens misc_RNA (LOC729406), miscRNA. |
| 1842 | ECM2 | -1.160 | extracellular matrix protein 2 |
| 643384 | LOC643384 | -1.161 | PREDICTED: Homo sapiens hypothetical LOC643384 (LOC643384), mRNA. |
| 3303 | HSPA1A | -1.162 | heat shock protein family A (Hsp70) member 1A |
| 10799 | RPP40 | -1.163 | ribonuclease P/MRP subunit p40 |
| 6513 | SLC2A1 | -1.164 | solute carrier family 2 member 1 |
| 55667 | DENND4C | -1.165 | DENN domain containing 4C |
| 3861 | KRT14 | -1.168 | keratin 14 |
| 7124 | TNF | -1.169 | tumor necrosis factor |
| 1808 | DPYSL2 | -1.170 | dihydropyrimidinase like 2 |
| 8581 | LY6D | -1.171 | lymphocyte antigen 6 family member D |
| 25941 | C18orf10 | -1.172 | tubulin polyglutamylase complex subunit 2 |
| 7277 | TUBA4A | -1.172 | tubulin alpha 4a |
| 2633 | GBP1 | -1.173 | guanylate binding protein 1 |
| 3988 | LIPA | -1.174 | lipase A, lysosomal acid type |
| 7347 | UCHL3 | -1.175 | ubiquitin C-terminal hydrolase L3 |
| 653888 | LOC653888 | -1.177 | PREDICTED: Homo sapiens similar to Actin-related protein 2/3 complex subunit 1B (ARP2/3 complex 41 kDa subunit) (p41-ARC) (LOC653888), mRNA. |
| 9303 | SNORD25 | -1.179 | small nucleolar RNA, C/D box 25 |
| 4723 | NDUFV1 | -1.190 | NADH:ubiquinone oxidoreductase core subunit V1 |
| 3326 | HSP90AB1 | -1.190 | heat shock protein 90 alpha family class B member 1 |
| 858 | CAV2 | -1.193 | caveolin 2 |
| 9086 | EIF1AY | -1.196 | eukaryotic translation initiation factor 1A, Y-linked |
| 978 | CDA | -1.197 | cytidine deaminase |
| 1382 | CRABP2 | -1.204 | cellular retinoic acid binding protein 2 |
| 60481 | ELOVL5 | -1.223 | ELOVL fatty acid elongase 5 |
| 7447 | VSNL1 | -1.226 | visinin like 1 |
| 4242 | MFNG | -1.229 | MFNG O-fucosylpeptide 3-beta-N-acetylglucosaminyltransferase |
| 400578 | LOC400578 | -1.233 | PREDICTED: Homo sapiens similar to Keratin, type I cytoskeletal 14 (Cytokeratin-14) (CK-14) (Keratin-14) (K14) (LOC400578), mRNA. |
| 375791 | C9orf169 | -1.238 | cysteine rich tail 1 |
| 7272 | TTK | -1.241 | TTK protein kinase |
| 1830 | DSG3 | -1.242 | desmoglein 3 |
| 6876 | TAGLN | -1.242 | transgelin |
| 26472 | PPP1R14B | -1.246 | protein phosphatase 1 regulatory inhibitor subunit 14B |
| 730107 | LOC730107 | -1.251 | PREDICTED: Homo sapiens similar to Glycine cleavage system H protein, mitochondrial (LOC730107), mRNA. |
| 51734 | SEPX1 | -1.252 | methionine sulfoxide reductase B1 |
| 114569 | MAL2 | -1.258 | mal, T-cell differentiation protein 2 (gene/pseudogene) |
| 51706 | CYB5R1 | -1.262 | cytochrome b5 reductase 1 |
| 163782 | ANKRD38 | -1.264 | KN motif and ankyrin repeat domains 4 |
| 80013 | FAM188A | -1.265 | MINDY lysine 48 deubiquitinase 3 |
| 6715 | SRD5A1 | -1.286 | steroid 5 alpha-reductase 1 |
| 4067 | LYN | -1.287 | LYN proto-oncogene, Src family tyrosine kinase |
| 5650 | KLK7 | -1.291 | kallikrein related peptidase 7 |
| 7045 | TGFBI | -1.294 | transforming growth factor beta induced |
| 55848 | C9orf46 | -1.296 | plasminogen receptor with a C-terminal lysine |
| 255488 | RNF144B | -1.297 | ring finger protein 144B |
| 8507 | ENC1 | -1.302 | ectodermal-neural cortex 1 |
| 23406 | COTL1 | -1.303 | coactosin like F-actin binding protein 1 |
| 501 | ALDH7A1 | -1.309 | aldehyde dehydrogenase 7 family member A1 |
| 51524 | TMEM138 | -1.311 | transmembrane protein 138 |
| 91523 | FAM113B | -1.312 | PC-esterase domain containing 1B |
| 200958 | MUC20 | -1.316 | mucin 20, cell surface associated |
| 94120 | SYTL3 | -1.322 | synaptotagmin like 3 |
| 84141 | TMEM166 | -1.324 | eva-1 homolog A, regulator of programmed cell death |
| 653506 | LOC653506 | -1.333 | PREDICTED: Homo sapiens similar to meteorin, glial cell differentiation regulator-like (LOC653506), mRNA. |
| 728910 | LOC728910 | -1.342 | PREDICTED: Homo sapiens similar to Galectin-7 (Gal-7) (HKL-14) (PI7) (p53-induced protein 1) (LOC728910), mRNA. |
| 1387 | CREBBP | -1.344 | CREB binding protein |
| 51442 | VGLL1 | -1.346 | vestigial like family member 1 |
| 857 | CAV1 | -1.348 | caveolin 1 |
| 440915 | FKSG30 | -1.350 | actin-like protein |
| 6662 | SOX9 | -1.351 | SRY-box 9 |
| 677800 | SNORA12 | -1.359 | small nucleolar RNA, H/ACA box 12 |
| 2729 | GCLC | -1.360 | glutamate-cysteine ligase catalytic subunit |
| 5909 | RAP1GAP | -1.363 | RAP1 GTPase activating protein |
| 2012 | EMP1 | -1.366 | epithelial membrane protein 1 |
| 5021 | OXTR | -1.375 | oxytocin receptor |
| 29984 | RHOD | -1.384 | ras homolog family member D |
| 10643 | IGF2BP3 | -1.391 | insulin like growth factor 2 mRNA binding protein 3 |
| 10597 | TRAPPC2P1 | -1.393 | trafficking protein particle complex 2B |
| 3887 | KRT81 | -1.395 | keratin 81 |
| 4828 | NMB | -1.400 | neuromedin B |
| 10486 | CAP2 | -1.400 | CAP, adenylate cyclase-associated protein, 2 (yeast) |
| 653499 | LOC653499 | -1.407 | Homo sapiens similar to Galectin-7 (Gal-7) (HKL-14) (PI7) (p53-induced protein 1) (LOC653499), mRNA. |
| 3852 | KRT5 | -1.412 | keratin 5 |
| 6319 | SCD | -1.414 | stearoyl-CoA desaturase |
| 1022 | CDK7 | -1.427 | cyclin dependent kinase 7 |
| 5010 | CLDN11 | -1.432 | claudin 11 |
| 55839 | CENPN | -1.433 | centromere protein N |
| 7991 | TUSC3 | -1.439 | tumor suppressor candidate 3 |
| 4499 | MT1M | -1.442 | metallothionein 1M |
| 1396 | CRIP1 | -1.443 | cysteine rich protein 1 |
| 55612 | FERMT1 | -1.447 | fermitin family member 1 |
| 26519 | TIMM10 | -1.447 | translocase of inner mitochondrial membrane 10 |
| 27076 | LYPD3 | -1.449 | LY6/PLAUR domain containing 3 |
| 54578 | UGT1A6 | -1.456 | UDP glucuronosyltransferase family 1 member A6 |
| 10808 | HSPH1 | -1.457 | heat shock protein family H (Hsp110) member 1 |
| 57001 | ACN9 | -1.459 | succinate dehydrogenase complex assembly factor 3 |
| 9388 | LIPG | -1.460 | lipase G, endothelial type |
| 6347 | CCL2 | -1.465 | C-C motif chemokine ligand 2 |
| 2950 | GSTP1 | -1.469 | glutathione S-transferase pi 1 |
| 57402 | S100A14 | -1.472 | S100 calcium binding protein A14 |
| 9531 | BAG3 | -1.484 | BCL2 associated athanogene 3 |
| 220002 | CYBASC3 | -1.492 | cytochrome b561 family member A3 |
| 644945 | MGC102966 | -1.497 | Homo sapiens similar to Keratin, type I cytoskeletal 16 (CK-16) |
| 112849 | C14orf149 | -1.500 | trans-L-3-hydroxyproline dehydratase |
| 7164 | TPD52L1 | -1.510 | tumor protein D52 like 1 |
| 3321 | IGSF3 | -1.519 | immunoglobulin superfamily member 3 |
| 4199 | ME1 | -1.521 | malic enzyme 1 |
| 5699 | PSMB10 | -1.524 | proteasome subunit beta 10 |
| 9289 | GPR56 | -1.545 | adhesion G protein-coupled receptor G1 |
| 1289 | COL5A1 | -1.566 | collagen type V alpha 1 chain |
| 23682 | RAB38 | -1.567 | RAB38, member RAS oncogene family |
| 10381 | TUBB3 | -1.569 | tubulin beta 3 class III |
| 7431 | VIM | -1.578 | vimentin |
| 10630 | PDPN | -1.587 | podoplanin |
| 55655 | NLRP2 | -1.590 | NLR family pyrin domain containing 2 |
| 3963 | LGALS7 | -1.599 | lectin, galactoside-binding, soluble, 7 (galectin 7) |
| 79755 | ZNF750 | -1.601 | zinc finger protein 750 |
| 9918 | NCAPD2 | -1.603 | non-SMC condensin I complex subunit D2 |
| 100132673 | LOC100132673 | -1.634 | PREDICTED: Homo sapiens misc_RNA (LOC100132673), miscRNA. |
| 1001 | CDH3 | -1.642 | cadherin 3 |
| 84080 | C16orf48 | -1.656 | enkurin domain containing 1 |
| 57415 | C3orf14 | -1.662 | chromosome 3 open reading frame 14 |
| 6001 | RGS10 | -1.671 | regulator of G protein signaling 10 |
| 29113 | C6orf15 | -1.675 | chromosome 6 open reading frame 15 |
| 29948 | OKL38 | -1.684 | pregnancy-induced growth inhibitor (OKL38), transcript variant 1 |
| 154091 | SLC2A12 | -1.689 | solute carrier family 2 member 12 |
| 27242 | TNFRSF21 | -1.714 | TNF receptor superfamily member 21 |
| 119710 | C11orf74 | -1.718 | chromosome 11 open reading frame 74 |
| 3685 | ITGAV | -1.746 | integrin subunit alpha V |
| 999 | CDH1 | -1.748 | cadherin 1 |
| 2017 | CTTN | -1.752 | cortactin |
| 56241 | SUSD2 | -1.753 | sushi domain containing 2 |
| 23643 | LY96 | -1.770 | lymphocyte antigen 96 |
| 51523 | CXXC5 | -1.798 | CXXC finger protein 5 |
| 5621 | PRNP | -1.824 | prion protein |
| 26053 | AUTS2 | -1.837 | AUTS2, activator of transcription and developmental regulator |
| 9686 | VGLL4 | -1.839 | vestigial like family member 4 |
| 54626 | HES2 | -1.841 | hes family bHLH transcription factor 2 |
| 12 | SERPINA3 | -1.846 | serpin family A member 3 |
| 29099 | COMMD9 | -1.876 | COMM domain containing 9 |
| 8500 | PPFIA1 | -1.877 | PTPRF interacting protein alpha 1 |
| 256764 | WDR72 | -1.915 | WD repeat domain 72 |
| 221120 | ALKBH3 | -1.969 | alkB homolog 3, alpha-ketoglutaratedependent dioxygenase |
| 8743 | TNFSF10 | -1.982 | TNF superfamily member 10 |
| 65983 | GRAMD3 | -2.046 | GRAM domain containing 2B |
| 79156 | PLEKHF1 | -2.057 | pleckstrin homology and FYVE domain containing 1 |
| 3429 | IFI27 | -2.062 | interferon alpha inducible protein 27 |
| 26353 | HSPB8 | -2.070 | heat shock protein family B (small) member 8 |
| 26579 | MYEOV | -2.081 | myeloma overexpressed |
| 6624 | FSCN1 | -2.086 | fascin actin-bundling protein 1 |
| 55107 | TMEM16A | -2.093 | anoctamin 1, calcium activated chloride channel (ANO1), transcript variant 1 |
| 8910 | SGCE | -2.101 | sarcoglycan epsilon |
| 55466 | DNAJA4 | -2.130 | DnaJ heat shock protein family (Hsp40) member A4 |
| 8804 | CREG1 | -2.160 | cellular repressor of E1A stimulated genes 1 |
| 645638 | LOC645638 | -2.168 | PREDICTED: Homo sapiens misc_RNA (LOC645638), miscRNA. |
| 55107 | ANO1 | -2.189 | anoctamin 1 |
| 220064 | ORAOV1 | -2.201 | oral cancer overexpressed 1 |
| 54765 | TRIM44 | -2.206 | tripartite motif containing 44 |
| 8772 | FADD | -2.211 | Fas associated via death domain |
| 586 | BCAT1 | -2.240 | branched chain amino acid transaminase 1 |
| 653499 | LGALS7B | -2.256 | galectin 7 |
| 1535 | CYBA | -2.265 | cytochrome b-245 alpha chain |
| 81706 | PPP1R14C | -2.268 | protein phosphatase 1 regulatory inhibitor subunit 14C |
| 27122 | DKK3 | -2.273 | dickkopf WNT signaling pathway inhibitor 3 |
| 7057 | THBS1 | -2.305 | thrombospondin 1 |
| 2919 | CXCL1 | -2.343 | C-X-C motif chemokine ligand 1 |
| 9076 | CLDN1 | -2.372 | claudin 1 |
| 960 | CD44 | -2.428 | CD44 molecule (Indian blood group) |
| 894 | CCND2 | -2.484 | cyclin D2 |
| 1728 | NQO1 | -2.520 | NAD(P)H quinone dehydrogenase 1 |
| 3627 | CXCL10 | -2.531 | C-X-C motif chemokine ligand 10 |
| 644936 | LOC644936 | -2.677 | actin beta pseudogene |
| 84969 | C20orf100 | -2.737 | TOX high mobility group box family member 2 |
| 10232 | MSLN | -2.785 | mesothelin |
| 595 | CCND1 | -2.870 | cyclin D1 |
| 150696 | PROM2 | -2.877 | prominin 2 |
| 24147 | FJX1 | -2.999 | four jointed box 1 |
| 151306 | GPBAR1 | -3.045 | G protein-coupled bile acid receptor 1 |
| 25818 | KLK5 | -3.321 | kallikrein related peptidase 5 |
| 4071 | S100A8 | -3.498 | S100 calcium binding protein A8 |
|  | CDH2 | -3.687 | cadherin 2 |
| 9022 | CLIC3 | -3.730 | chloride intracellular channel 3 |
|  | TM4SF1 | -4.208 | transmembrane 4 L six family member 1 |
|  |  |  |  |
