# Supplementary material for: Comparative Gene Expression Profiling of Tobacco-Associated HPV-Positive versus Negative Oral Squamous Carcinoma Cell Lines
Source: Int J Med Sci. 2020 Jan 1;17(1):112–24. doi: 10.7150/ijms.35133 (PMC6945558; doi:10.7150/ijms.35133)
Supplement: Supplementary file 1 — Supplementary figures and tables. [file ijmsv17p0112s1.zip › Supplementary Table S2.docx]

**Supplementary Table S2.** Clinicopathological features of HNSCCs from GSE65858 dataset.

| **Samples** | **HPV-positive** | **HPV-negative** |
| --- | --- | --- |
| **N** | 42 | 170 |
| **Age, years** |  | |
| **Mean** (SD) | 59.5 (8.8) | 58.9 (9.8) |
| **Gender, n (%)** |  | |
| F | 8 (33.3) | 20 (33.3) |
| M | 34 (66.7) | 150 (66.7) |
| **Tumor Type, n (%)** |  | |
| Primary | 42 (100.0) | 157 (92.4) |
| Relapse | 0 (0.0) | 2 (1.2) |
| Secondary | 0 (0.0) | 11 (6.4) |
| **Tumor Site, n (%)** |  | |
| Cavum Oris | 8 (19.0) | 57 (33.5) |
| Hypopharynx | 1 (2.4) | 27 (15.9) |
| Larynx | 2 (4.8) | 39 (22.9) |
| Oropharynx | 31 (73.8) | 46 (27.1) |
| Unknown | 0 (0.0) | 1 (0.6) |
| **UICC Stage, n (%)** |  | |
| I | 1 (2.4) | 16 (9.4) |
| II | 5 (11.9) | 26 (15.3) |
| III | 7 (16.7) | 23 (13.5) |
| IVA | 21 (50) | 95 (56) |
| IVB | 7 (16.7) | 6 (3.5) |
| IVC | 1 (2.4) | 4 (2.3) |
| **T Category, n (%)** |  | |
| 1 | 6 (14.3) | 23 (13.5) |
| 2 | 16 (38.1) | 48 (28.2) |
| 3 | 6 (14.3) | 40 (23.5) |
| 4a | 10 (23.8) | 58 (34.1) |
| 4b | 4 (9.5) | 1 (0.6) |
| **N category, n(%)** |  | |
| 0 | 8 (19.0) | 69 (40.6) |
| 1 | 6 (14.3) | 18 (10.6) |
| 2a | 1 (2.4) | 6 (3.5) |
| 2b | 12 (28.6) | 37 (21.8) |
| 2c | 11 (26.2) | 33 (19.4) |
| 3 | 4 (9.5) | 7 (4.1) |
| **Treatment, n (%)** |  | |
| Monotherapy | 11 (26.2) | 51 (30.0) |
| Multidisciplinary treatment | 31 (73.8) | 118 (69.4) |
| Palliative treatment | 0 (0.0) | 1 (0.6) |
