# Supplementary material for: Comparative Gene Expression Profiling of Tobacco-Associated HPV-Positive versus Negative Oral Squamous Carcinoma Cell Lines
Source: Int J Med Sci. 2020 Jan 1;17(1):112–24. doi: 10.7150/ijms.35133 (PMC6945558; doi:10.7150/ijms.35133)
Supplement: Supplementary file 1 — Supplementary figures and tables. [file ijmsv17p0112s1.zip › Supplementary Table S3.docx]

**Supplementary Table S3**. Molecular characteristics of analyzed cell lines.

| CELL LINE | Gender | Age at diagnosis (years) | Ethnicity | Smoking | Primary tumor size | TP53 | HPV+ |
| --- | --- | --- | --- | --- | --- | --- | --- |
| BICR22 | M | 88 | Caucasian | yes | Tongue | Mutated | Negative |
| BICR31 | M | Unknow | Caucasian | yes | Tongue | Mutated | Negative |
| CAL27 | M | 56 | Caucasian | yes | Tongue | Mutated | Negative |
| DOK | M | 57 | Caucasian | yes | Oral Cavity | Mutated | Negative |
| H103 | M | 32 | Caucasian | yes | Tongue | Mutated | Negative |
| H357 | M | 74 | Caucasian | yes | Tongue | Mutated | Negative |
| H400 | F | 55 | Unknown | yes | Alveolar Process | Mutated | Negative |
| HN6 | M | 54 | Unknown | yes | Tongue | Mutated | Negative |
| MSK921 | Unknow | Unknow | Unknown | yes | Tongue | Wild Type | Negative |
| OECM1 | M | Unknow | Chinese | yes | Oral Cavity Gingival | Mutated | Negative |
| SCC68 | M | Unknow | Unknown | yes | Oral Cavity | Wild Type | Negative |
| SCC47 | M | 53 | Unknown | yes | Tongue | Wild Type | Positive |
| SCC90 | M | Unknow | Unknown | yes | Upper Aerodigestive Tract | Wild Type | Positive |
